# Identification of two heterogeneous subtypes of hepatocellular carcinoma with distinct pathway activities and clinical outcomes based on gene set variation analysis

**DOI:** 10.3389/fgene.2024.1441189

**Published:** 2024-09-10

**Authors:** Zhipeng Jin, Xin Wang, Xue Zhang, Siqi Cheng, Yefu Liu

**Affiliations:** ^1^ Department of Hepatopancreatobiliary Surgery, Liaoning Cancer Hospital and Institute, Shenyang, Liaoning, China; ^2^ Central Laboratory, Liaoning Cancer Hospital and Institute, Shenyang, Liaoning, China

**Keywords:** hepatocellular carcinoma (HCC), molecular subtype, gene set variant analysis, gene signature, prognosis, tumor microenvironment, RFPL4B

## Abstract

**Background:**

High heterogeneity is an essential feature of malignant tumors. This study aims to reveal the drivers of hepatocellular carcinoma heterogeneity for prognostic stratification and to guide individualized treatment.

**Methods:**

Omics data and clinical data for two HCC cohorts were derived from the Cancer Genome Atlas (TCGA) and the International Cancer Genome Atlas (ICGC), respectively. CNV data and methylation data were downloaded from the GSCA database. GSVA was used to estimate the transcriptional activity of KEGG pathways, and consensus clustering was used to categorize the HCC samples. The pRRophetic package was used to predict the sensitivity of samples to anticancer drugs. TIMER, MCPcounter, quanTIseq, and TIDE algorithms were used to assess the components of TME. LASSO and COX analyses were used to establish a prognostic gene signature. The biological role played by genes in HCC cells was confirmed by *in vitro* experiments.

**Results:**

We classified HCC tissues into two categories based on the activity of prognostic pathways. Among them, the transcriptional profile of cluster A HCC is similar to that of normal tissue, dominated by cancer-suppressive metabolic pathways, and has a better prognosis. In contrast, cluster B HCC is dominated by high proliferative activity and has significant genetic heterogeneity. Meanwhile, cluster B HCC is often poorly differentiated, has a high rate of serum AFP positivity, is prone to microvascular invasion, and has shorter overall survival. In addition, we found that mutations, copy number variations, and aberrant methylation were also crucial drivers of the differences in heterogeneity between the two HCC subtypes. Meanwhile, the TME of the two HCC subtypes is also significantly different, which offers the possibility of precision immunotherapy for HCC patients. Finally, based on the prognostic value of molecular subtypes, we developed a gene signature that could accurately predict patients’ OS. The riskscore quantified by the signature could evaluate the heterogeneity of HCC and guide clinical treatment. Finally, we confirmed through *in vitro* experiments that RFPL4B could promote the progression of Huh7 cells.

**Conclusion:**

The molecular subtypes we identified effectively exposed the heterogeneity of HCC, which is important for discovering new effective therapeutic targets.

## 1 Introduction

Hepatocellular carcinoma is the most common type of malignancy of the liver, accounting for about 85%–90% of all cases (Sartorius et al., 2015). The regions with the highest incidence and mortality of HCC are East Asia and Africa, where chronic HBV infection and AFB1 exposure are the major risk factors (McGlynn et al., 2015). In fact, cirrhosis from any etiology is the strongest risk factor for HCC, which also includes chronic alcohol consumption, diabetes, or obesity-related non-alcoholic steatohepatitis, and infection by HCV, etc (Marrero et al., 2018). Currently, the choice of optimal treatment strategy for HCC is based on the tumor stage. For early-stage HCC, ablation and surgery are the main treatment modalities. For intermediate and advanced HCC, chemoembolization and systemic therapy are preferred. In recent years, great breakthroughs have been made in systemic therapy as a focus of research. In particular, the use of multi-kinase inhibitors, anti-angiogenic drugs and immune checkpoint inhibitors has led to improvements in the prognosis of patients with HCC. However, HCC remains one of the worst human malignancies in terms of clinical outcome, with a median OS of less than 2 years for patients with advanced stage (Llovet et al., 2021).

The high heterogeneity, including genetic and immunological heterogeneity, is one of the main reasons for the poor treatment response of HCC (Kelley, 2015; Sia et al., 2017). Therefore, it is of paramount importance to deeply characterize the tumor and implement precise and personalized treatment. Since the 21st century, the development of high-throughput sequencing technologies has made it possible to analyze the transcriptome and genome of a species in a detailed and holistic manner. At the beginning of the 21st century, many studies of high-throughput data from HCC samples have provided us with a better understanding of HCC (Boyault et al., 2007; Hoshida et al., 2009; Lee et al., 2004; Chiang et al., 2008). The later TCGA, ICGC, and CPTAC projects were more comprehensive and in-depth studies of genomic heterogeneity in HCC from a multi-omics perspective (Cancer Genome Atlas Research Network, 2017; Zhang et al., 2019; Gao et al., 2019). Unsurprisingly, many of the molecular features of HCC have gradually been uncovered, and these features also divide HCC into two main subclasses: the proliferation class and the non-proliferation class. These two subclasses of HCC differ significantly in many ways, including aetiology, molecular characteristics, prognosis, clinical indicators, and so on (Llovet et al., 2021). Unfortunately, these findings have not yet been successfully applied in clinical practice, making it still difficult to achieve precision and personalization in the systemic treatment of HCC. In fact, the functional pathways and molecular networks behind HCC are complex. Previous studies mainly focused on a few typical pathways, so there is still a long way to go to fully dissect the heterogeneity of HCC.

In this study, we first screened for prognosis-related pathways by GSVA and classified all HCC samples into two subclasses based on the activity of these pathways. One of the two subclasses is characterized by high metabolic activity similar to that of normal samples and is referred to as the metabolic-dominant subtype, which has a relatively better prognosis. In contrast, the gene expression profile of another subtype differs considerably from that of non-cancerous tissues and is mainly characterized by higher activity of pathways such as the cell cycle and genetic information processing, and lower activity of those metabolic pathways associated with good prognosis. We refer to this type of HCC, which has a relatively worse prognosis, as the proliferation-dominant subtype. Then, we compared the clinicopathological and genomic characteristics of the two subtypes of HCC and predicted their response to anti-cancer drug therapy. Finally, we constructed a gene risk scoring system to predict molecular subtypes, overall survival, and drug sensitivity. Our findings were highly consistent across two large sample HCC cohorts and the conclusion of this study may have potential guidance for the personalized treatment of HCC.

## 2 Materials and methods

**FIGURE 1:**
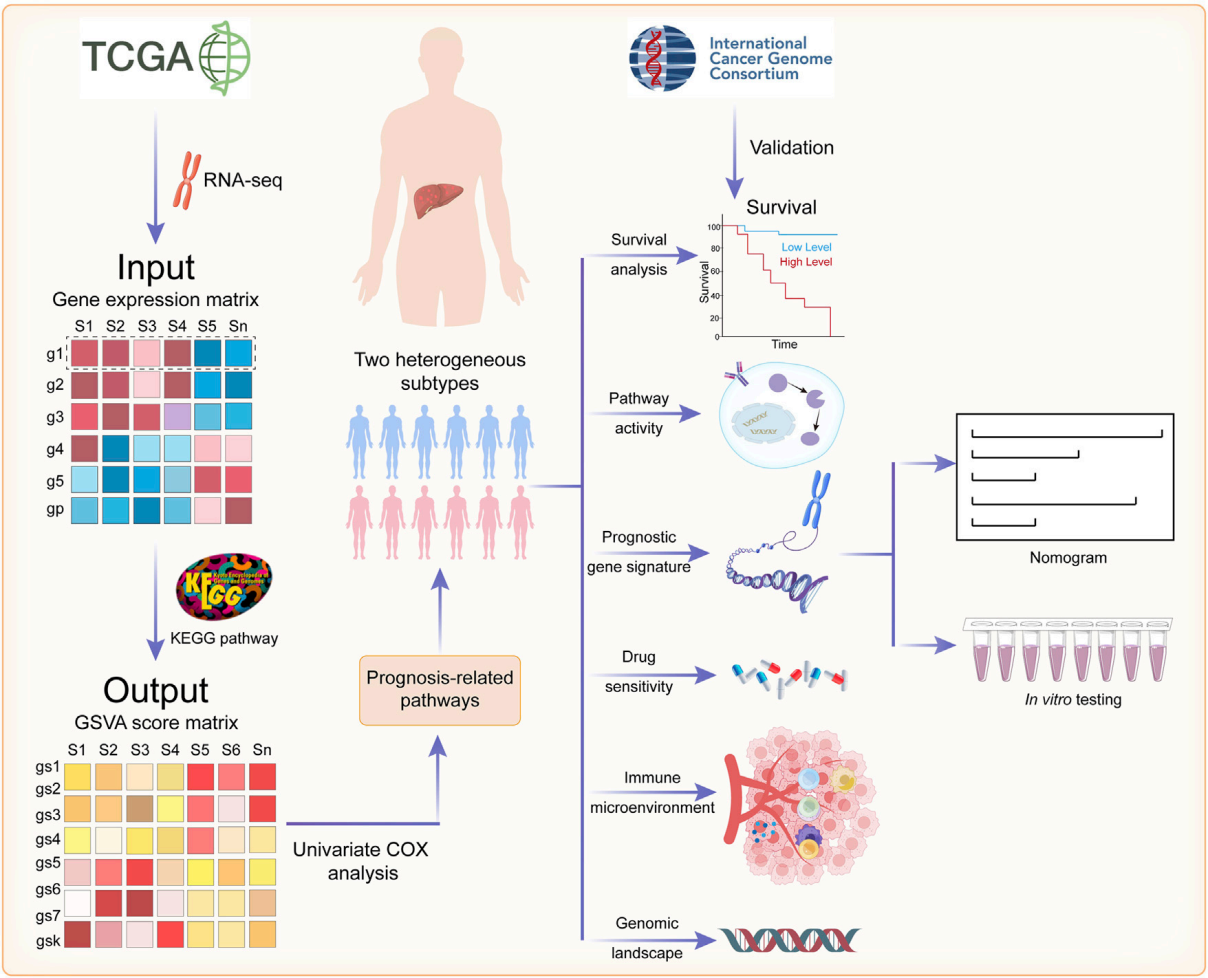
The flowchart of the present study.

The flowchart of this study is shown in Figure 1.

### 2.1 Dataset sources and preprocessing

RNA-seq data, clinical data, and somatic mutation data from two HCC cohorts were analyzed in this study. The TCGA cohort (normal n = 50, HCC n = 374) was downloaded from the GDC Data Portal (https://portal.gdc.cancer.gov/). The ICGC cohort (normal n = 202, HCC n = 243) was downloaded from the ICGC Data Portal (https://dcc.icgc.org/). TPM values were used as normalized gene expression for subsequent analysis.

### 2.2 Identification of HCC subclasses

Based on the gene expression data, gene set variation analysis was performed using the *GSVA* package to obtain the activity of 186 KEGG pathways for each HCC sample (Hanzelmann et al., 2013). The annotation file (c2.cp.kegg.v7.5.1.symbols.gmt) was downloaded from the MSigDB (http://www.gsea-msigdb.org/gsea/downloads.jsp). The categories for each pathway were obtained from the KEGG PATHWAY Database (https://www.kegg.jp/kegg/pathway.html). Univariate COX regression analysis was used to screen prognosis-related KEGG pathways. Then, consensus clustering was performed to identify prognostic subclasses of HCC samples based on the ES of pathways with prognostic value. The *ConsensuClusterPlus* package was applied to determine the most stable clustering (Wilkerson and Hayes, 2010). Principal component analysis (PCA) between the two subclasses was performed using the *ggplot2* package and the ES of pathways was visualized using the *pheatmap* package.

### 2.3 Comparison of genomic features of HCC subclasses

Somatic mutation data (MAF file) was visualized by maftools package (Mayakonda et al., 2018). The TMB for each HCC sample was calculated from the somatic mutation data. CNV data and methylation data of TCGA cohort were downloaded from GSCA database (http://bioinfo.life.hust.edu.cn/GSCA/#/) (Liu et al., 2018). The CNV data was processed through GISTIC2.0 method (Mermel et al., 2011). As for the methylation data, GSCA screened for the CpG site most negatively correlated with the corresponding gene expression for subsequent analysis. In our study, to ensure significant differences and pathway enrichment, we considered CpG sites with |Δ mean β-value| >0.1 and *FDR* < 0.05 as methylation variable positions. Subsequently, KEGG enrichment analysis was performed to explore the pathways enriched by genes with differential copy number or methylation level between two HCC subclasses.

### 2.4 Construction and validation of the subclass-related prognostic mRNA signature

First, differentially expressed mRNAs (DEmRNAs) between the two prognostic subclasses were identified by the *limma* package. The threshold for screening was set at |log2 Fold Change| > 1 and *FDR* < 0.05. Then, univariate Cox regression analysis was performed using the *survival* package to assess the prognostic value of mRNAs and the threshold was set at *P* < 0.001. Next, LASSO regression was used to reduce the dimensionality of prognosis-related differentially expressed mRNAs by using *glmnet* package. Finally, multivariable Cox regression analysis was used to select the candidate mRNAs and establish an optimal prognostic signature, the riskscore. The riskscore was calculated as follows: riskscore = Σexpi * coefi. In the TCGA cohort, we included age, gender, tumor stage and tumor grade as confounding factors for the subsequent analysis. Therefore, we here excluded the patients who lacked complete clinical information on all four of above. In addition, the entire TCGA cohort was randomly divided into training (n = 171) and testing (n = 171) cohorts and the former was used to construct the signature. The testing cohort and the ICGC cohort were used to validate the signature. In each cohort, all patients were also divided into high-risk and low-risk groups based on the median value of the riskscores. The predictive performance of the riskscore was evaluated by time-dependent ROC curves.

### 2.5 Development and validation of a clinical nomogram

In the training cohort, the clinical characteristics and riskscore were used to develop a nomogram using the *rms* package based on the independent prognostic factors from the result of the multivariable Cox regression analysis. The predictive performance of the nomogram was evaluated by time-dependent ROC curves, calibration curves and C-index in the training cohort and two validation cohorts.

### 2.6 Prediction of sensitivity of HCC samples to anti-cancer drugs

In this study, we predicted the sensitivity of HCC samples to anti-cancer drugs. The 50% inhibitory concentration (IC50) was used as an indicator of sensitivity. Based on the gene expression data, the estimated IC50 of cancer samples to drugs was quantified by the *pRRophetic* package (Geeleher et al., 2014). The version of the data used for the analysis was CGP2016, which contains a total of 251 anti-cancer drugs.

### 2.7 Assessment of the TME in HCC samples

A previous study conducted an extensive immunogenomic analysis of over 10,000 tumor samples in the TCGA and identified six immune subtypes span cancer tissue types (Thorsson et al., 2019). Here, we first compared the distribution of HCC samples in the TCGA cohort between cancer immune subtypes and our prognostic HCC subclasses. Then, we used three independent methods, TIMER (Li et al., 2016), MCPcounter (Becht et al., 2016) and quanTIseq (Finotello et al., 2019), to predict the abundance of different immune cell types in HCC tissues. The analysis was performed with the *IOBR* package (Zeng et al., 2021). Finally, we used the TIDE (Jiang et al., 2018) algorithm and ImmuCellAI (Miao et al., 2020) algorithm to predict the response of HCC tissues to immunotherapy.

### 2.8 Identification of pathways associated with riskscore

We used gene set enrichment analysis (GSEA) (Subramanian et al., 2005) to identify the KEGG pathways that were closely associated with the riskscore based on the gene expression array. The analysis was performed using GSEA software V4.2.1 (http://www.gsea-msigdb.org/gsea/index.jsp). The annotation file (c2.cp.kegg.v7.5.1.symbols.gmt) was used as the reference and the permutation number was set to 1,000. Pathways with *FDR* < 0.5 and normalized enrichment score (NES) > 1.5 were considered to be significantly enriched.

### 2.9 Collection of the clinical specimens

The clinical specimens involved in this study were obtained from HCC patients who underwent hepatectomy at Liaoning Cancer Hospital. All patients had been pathologically confirmed and diagnosed with HCC. Cancer tissues and matched paracancerous tissue were frozen immediately in liquid nitrogen after resection and then stored at −80°C prior to use. The study was approved by the Ethics Committee of the Liaoning Cancer Hospital and conducted following the Declaration of Helsinki.

### 2.10 Cell culture and transfection

Human HCC-derived Huh7 cell was obtained from the American Type Culture Collection (ATCC). Cells were cultured with DMEM (Gibco, Life Technologies, USA) with 10% FBS (Gibco, Life Technologies, United States) and 1% Penicillin-Streptomycin at 37°C in a humidified incubator with5% CO_2_ atmosphere.

*In vitro* growing cell lines were treated with small interfering RNA (siRNA) against RFPL4B (Sangon Biotech, China) genes and si-NC (Sangon Biotech, China), according to the manufacturer’s recommendations, and incubated for 24 and 48 h. The siRNA sequences are listed in Supplementary Table S1.

### 2.11 Real-time quantitative PCR

Primers for human RFPL4B and GAPDH genes were purchased from Sangon Biotech (Shanghai, China), and the sequences were listed in Supplementary Table S2. PCR reactions were performed with 100 ng of cDNA, using a Rotor-Gene^®^-Q real-time PCR cycler (Roche LightCycler 96) and TaqMan Universal PCR Master Mix (Applied Biosystems). Cycling conditions were: 10 min of denaturation at 95°C and 40 cycles at 95°C for 15 s and at 60°C for 1 min. The relative transcription levels of the genes were calculated using the delta-delta-Ct (ΔΔCT) method (expressed as 2−^ΔΔCT^) and normalized to GAPDH as an endogenous control.

### 2.12 Cell viability analysis

To assess cell viability, Huh7 cell lines were plated at the concentration of 2.0 × 10^3^/well in 96-well plates, allowed to attach and adjust for the next 12 h and grown for the additional 24 and 48 h. The viability was assessed at these three time points - 0, 24, and 48 h with the cck-8 (MCE, USA) by measuring the absorbance at 450 nm after 1 h of incubation.

### 2.13 Migration and invasion assays

Transwell 1-10 × 10^4^ cells were plated in the upper chambers with Matrigel coated to estimate tumor invasion, and the chambers without Matrigel were used to assess tumor cell migration. In a 24-well plate, the upper wells were added with 200 μL serum-free medium, and the lower wells were added with 800 μL medium containing 10% FBS. The cells were incubated for 24–48 h. After Huh7 cells were completely attached to the wall, si-NC or si-RFPL4B was transfected with a final concentration of 100 nM. At the observation time point, the cells were cleared from the surface of the upper chambers’ membrane with a cotton swab. The invasive/migratory cells were fixed with 4% paraformaldehyde and stained by 0.1% crystal violet. The quantity of cells was calculated in 5 different areas under a microscope.

### 2.14 Wound healing migration assay

After Huh7 cells were completely attached, si-RFPL4B was transfected with a final concentration of 100 nM. After the cells were attached to the wall, the cells were scribed with a 200 μL tip, and the horizontal and vertical lines were scribed three times in each well. Make sure the force is uniform and the tip of the gun is perpendicular. Wash out the detached cells with preheated PBS, add 2 mL of culture medium (0%–3% FBS) into each well and continue to incubate. Record at 0 and 48 h respectively, and take pictures of the scratches. ImageJ software was used to measure the area of cell scratches, and the wound healing rate was used to reflect the cell migration ability.

### 2.15 Flow cytometry

Resuspend 1 × 10^6^ cells, centrifuge at 1,000 g for 5 min, discard supernatant, add Annexin-V/PI conjugate to gently resuspend cells, incubate at room temperature away from light for 20 min, followed by placing in an ice bath. Followed by flow cytometry. Propidium iodide has excitation (535 nm) and emission (595 nm) wavelengths and was detected using the PE channel. Similarly, Annexin-V has excitation (485 nm) and emission (535 nm) wavelengths, and its fluorescence was detected using the FITC channel. The data were analyzed using FACSDiva Version 6.1.3.

### 2.16 Statistical analysis

All statistical analyses were performed using R software (V4.1.2). Comparisons between groups were presented via Wilcoxon rank-sum test, Kruskal-Wallis test and ANOVA test. The diagnostic value of variables was evaluated by ROC curves. The correlation between variables was measured by Spearman correlation test and Chi-Squared test. K-M method and log-rank test were utilized to compare survival differences between groups of patients. Univariate COX regression was used to assess the prognostic value of continuous variables and multivariate COX regression was used to perform independent prognostic analysis and to construct prognostic models. The optimal model was identified based on the Akaike information criterion (AIC). *P-value* < 0.05 was considered statistically significant unless otherwise specified.

## 3 Results

### 3.1 HCC samples were divided into two prognosis-related molecular subclasses

**FIGURE 2:**
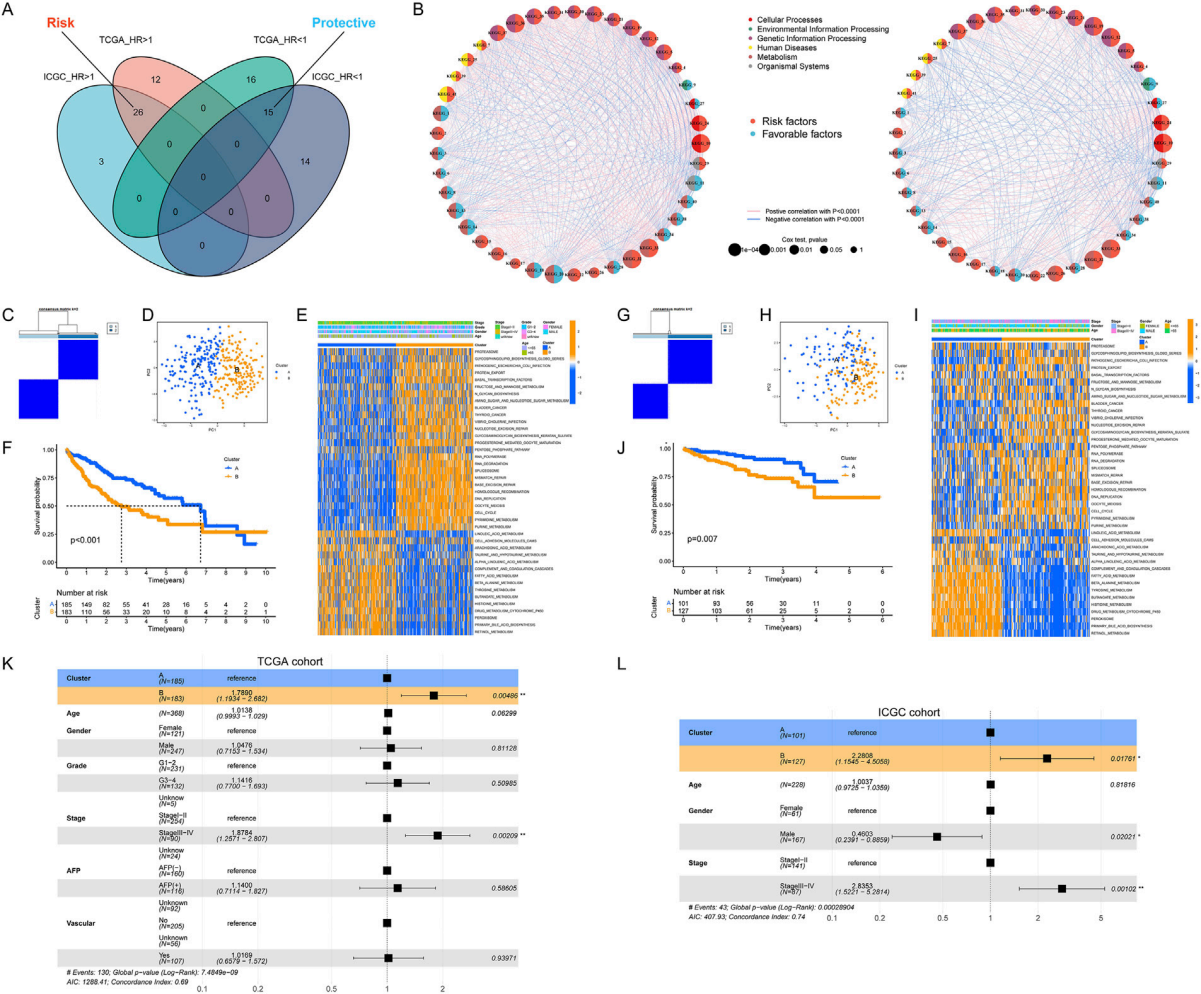
Identification of HCC molecular subtypes. **(A)** Identification of prognosis-related pathways in HCC; **(B)** Correlation between the activity of prognosis-related pathways; **(C, D)** Classification of HCC samples according to pathway activity; **(E)** Heatmap of pathway activity in each HCC subtype; **(F)** Significant differences in OS in patients with different HCC subtypes; **(G–J)** Validation of HCC classification in the ICGC cohort; **(K, L)** Molecular subtype as a prognostic risk factor for OS in HCC patients.

First, based on the GSVA scores, we evaluated the prognostic value of KEGG pathways. As shown in Figure 2A, a total of 26 risk pathways and 15 protective pathways showed prognostic value in both the TCGA and ICGC cohorts (Supplementary Table S3). The results in Figure 2B showed that there were consistent correlations between these pathways in both two cohorts. Moreover, the risk pathways were mostly related to genetic information processing and cellular processes, while the protective pathways were mostly related to metabolism. Thus, the molecular characteristics of HCC appeared to be used to predict prognosis. Next, we used consensus clustering to categorize the HCC samples based on the activity of prognosis-related pathways. As shown in Figure 2C, all HCC samples in TCGA cohort could be readily divided into two subclasses named cluster A and cluster B. The results of PCA and heatmap also revealed significant differences in transcriptional patterns between the two subclasses (Figures 2D, E). As expected, the OS in patients with cluster A was longer than that in patients with cluster B (Figure 2F). And the clustering results were verified in ICGC cohort (Figures 2G–J). Interestingly, the transcriptional profile of cluster A was similar to that of normal tissue, whereas cluster B was significantly different from normal tissue (Supplementary Figure S1). Then, we compared the differences in clinicopathological parameters between two subclasses. The results showed that, compared to cluster A, cluster B has the following characteristics: poorer tumor differentiation, more advanced stage, younger age, more female patients, more prone to vascular invasion and higher serum AFP levels (Supplementary Figure S2). Finally, we conducted multivariate Cox regression analysis and found that our clustering could also be used independently of clinicopathological parameters as an independent risk factor for OS in HCC patients (Figures 2K, L).

### 3.2 Distinct genomic profiles existed between two HCC subclasses

**FIGURE 3:**
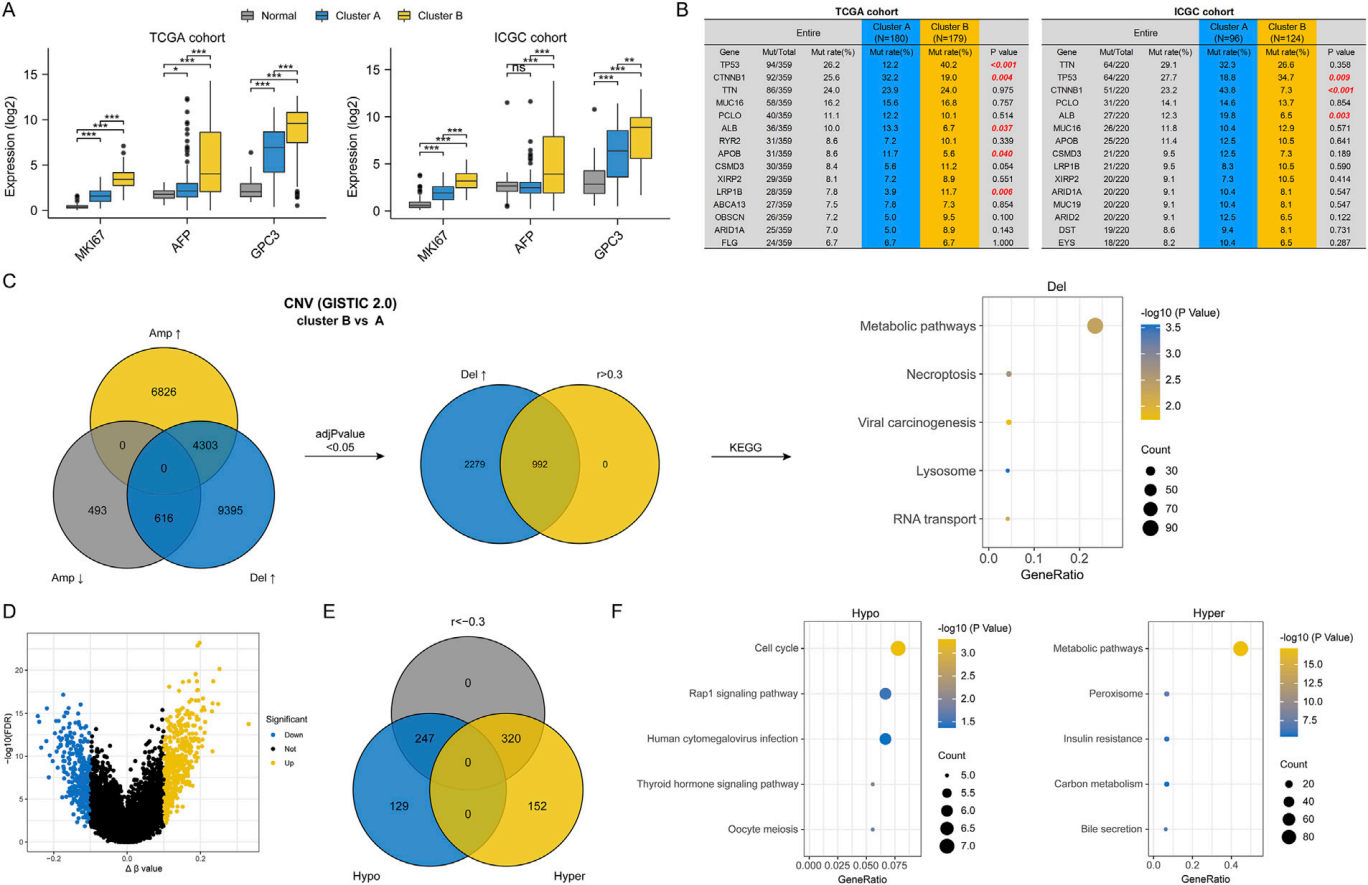
Differences in genomic characterization between two HCC subtypes. **(A)** Differences in marker gene expression in different HCC subtypes; **(B)** Differences in the frequency of mutations in different HCC subtypes; **(C)** Differences in CNVs between two HCC subtypes and pathways driven by CNVs; **(D)** Volcano plot of differentially methylated genes between two HCC subtypes; **(E)** Venn diagram of differentially methylated genes; **(F)** Pathways driven by differential DNA methylation. (ns, no statistical signifcance, *P < 0.05, **P < 0.01, and ***P < 0.001).

First, we compared the transcriptional expression of three most commonly used biomarkers of HCC (MKI67, AFP, and GPC3), all of which are thought to be associated with poor prognosis. As expected, their expression was higher in HCC tissues than in normal tissues and was also higher in cluster B than in cluster A (Figure 3A). Next, we analyzed the mutation frequency of the top fifteen genes with the highest mutation frequency in each HCC cohort. In TCGA cohort, there are different mutational profiles between two HCC subclasses (Supplementary Figure S3A). In detail, compared to cluster A, cluster B had higher mutation frequencies of TP53 and LRP1B, and lower mutation frequencies of CTNNB1, ALB and APOB. While differences in mutation frequencies of TP53, CTNNB1 and ALB were also present in the ICGC cohort (Figure 3B). However, there was no significant difference in TMB between two HCC subclasses (Supplementary Figure S3B). Then, we analyzed the CNV data of HCC samples in TCGA cohort. As shown in Figure 3C, cluster B had a higher frequency of CNV. In cluster B, 4,303 genes had increased amplification and deletion rates, and 616 genes had decreased amplification and increased deletion rates. Interestingly, we did not identify any genes with decreased deletion rates in cluster B compared to cluster A. We used the bonferroni method to adjust *P-value* and thus screened for genes with significantly different CNV frequencies between the two HCC subclasses and a significant positive correlation (r > 0.3) between CNV and gene expression. As a result, 992 genes with an increased frequency of deletion in cluster B were identified and enrichment analysis for these genes showed that they were closely associated with metabolic pathways. This suggested that the low activity of metabolic pathways in cluster B was related to the deletion in copy numbers of metabolism-related genes. However, after adjusting the *P-value*, we did not identify any genes with significantly different amplification frequencies between the two subclasses. Finally, we used a similar approach to analyse the potential effect of DNA methylation levels on the activity of pathways in HCC. The results in Figures 3D–F showed that high activity of cell cycle and low activity of metabolic pathways in cluster B might be due in part to regulation by DNA methylation.

### 3.3 Differences in drug sensitivity between two HCC subclasses

**FIGURE 4:**
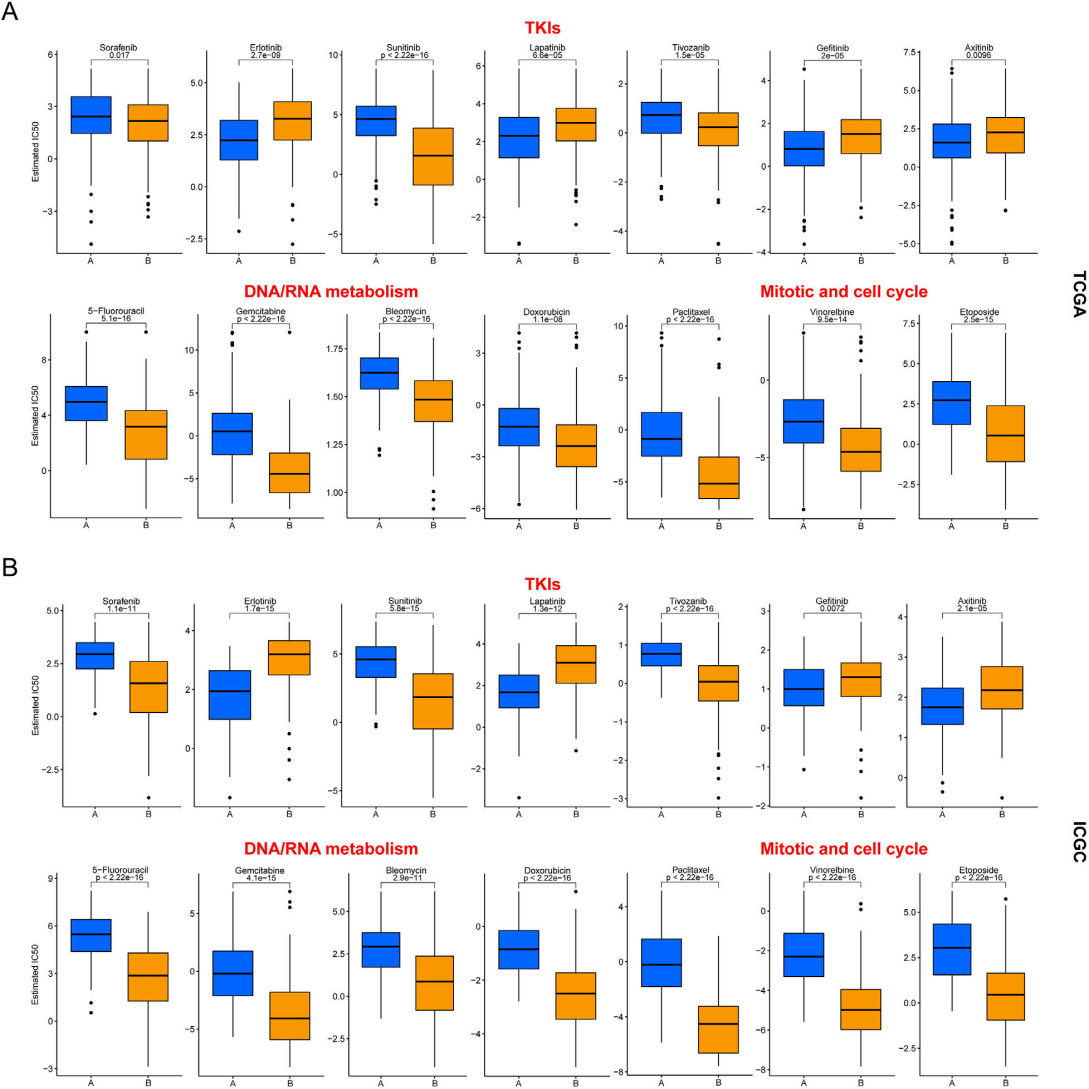
Differences in drug sensitivity between two HCC subclasses. **(A)** Results in the TCGA cohort; **(B)** Results in the ICGC cohort.

To further utilize our clustering to guide the clinical precision medicine for HCC, we analyzed the differences in anti-cancer drug sensitivity between the two subclasses. Here, we selected several drugs that are commonly chosen in anti-cancer treatment, including sorafenib, which has long been the first-line treatment option for unresectable HCC. Depending on the therapeutic mechanism of the drugs, they can be divided into three main categories: multi-tyrosine kinase inhibitors (TKIs), nucleic acid metabolism inhibitors (including 5-fluorouracil, gemcitabine, bleomycin, and doxorubicin) and cell proliferation inhibitors (including paclitaxel, vinorelbine, and etoposide). The results in Figure 4A showed that cluster B was more sensitive to nucleic acid metabolism inhibitors and cell proliferation inhibitors, all of which are chemotherapeutic agents. The above conclusion was consistent with the transcriptional profile of cluster B. As for the TKIs, cluster B was more sensitive to sorafenib, sunitinib and tivozanib, while the opposite was true for erlotinib, lapatinib, gefitinib and axitinib. Furthermore, the above differences were also verified in the ICGC cohort (Figure 4B). Thus, our clustering has the potential to guide the clinical precision medicine for HCC.

### 3.4 Differences in TME between two HCC subclasses

**FIGURE 5:**
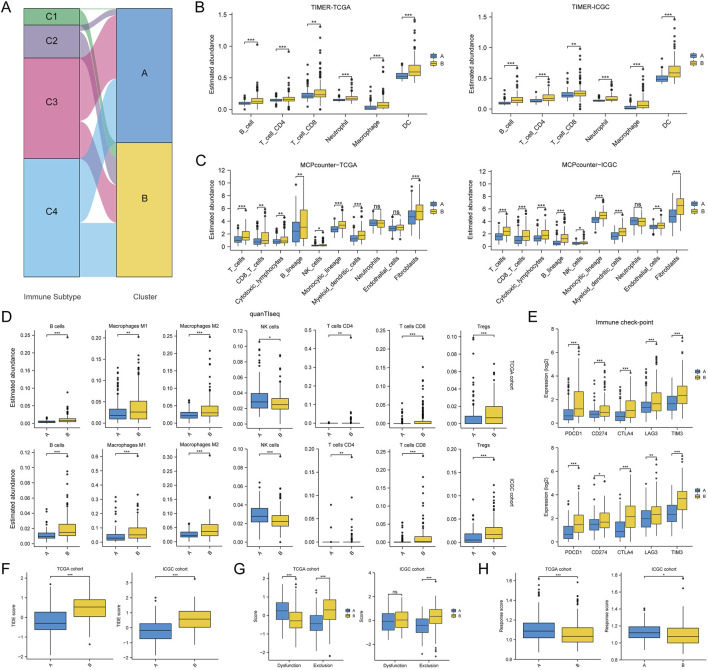
Differences in tumor immune microenvironment between two HCC subtypes. **(A)** Association of HCC classification with tumor immune subtypes; **(B–D)** Differences in the abundance of various immune cell types between two HCC subtypes assessed using TIMER, MCPcounter and quanTIseq algorithms; **(E)** Differences in immune checkpoint gene expression between two HCC subtypes; **(F)** Differences in TIDE score between two HCC subtypes; **(G)** Differences in dysfunction score and exclusion score calculated by the TIDE algorithm between the two HCC subtypes; **(H)** Differences in response score calculated by the ImmuCell AI algorithm between the two HCC subtypes. (ns, no statistical signifcance, *P < 0.05, **P < 0.01, and ***P < 0.001).

Given the critical nature of TME for cancer development and progression, we also compared the differences in TME between two HCC subclasses. The results in Figure 5A showed a correlation between our clustering and the cancer immune subtype. In detail, the C1 and C2 immune subtypes with high proliferation activity and high heterogeneity mostly belonged to cluster B, the C3 immune subtype with low proliferation activity and low heterogeneity was mostly cluster A, and the C4 immune subtype with moderate proliferation activity and moderate heterogeneity was independent of our clustering. In addition, the results of three independent immune cell infiltration prediction algorithms all showed differences in the abundance of multiple immune cell types between two HCC subclasses (Figures 5B–D). Although the prediction results of quanTIseq algorithm for NK cells differed from the other two algorithms, there appeared to be a higher level of immune cell abundance in cluster B. Also, the expression of six common immune checkpoint genes in cluster B was higher than in cluster A (Figure 5E). This led us to wonder if cluster B was more likely to benefit from immune checkpoint inhibitor therapy. However, cluster B had a higher median TIDE score, meaning it was less responsive to ICI treatment (Figure 5F). The potential reason for this might be that immune cells in cluster B tissues were unable to successfully infiltrate the tumor and were thus excluded from the cancerous tissues (Figure 5G). Meanwhile, the results of ImmuCell AI algorithm also reaffirmed that cluster B may respond less well to ICI treatment (Figure 5H).

### 3.5 Our clustering correlated with previous HCC molecular subtypes

**FIGURE 6:**
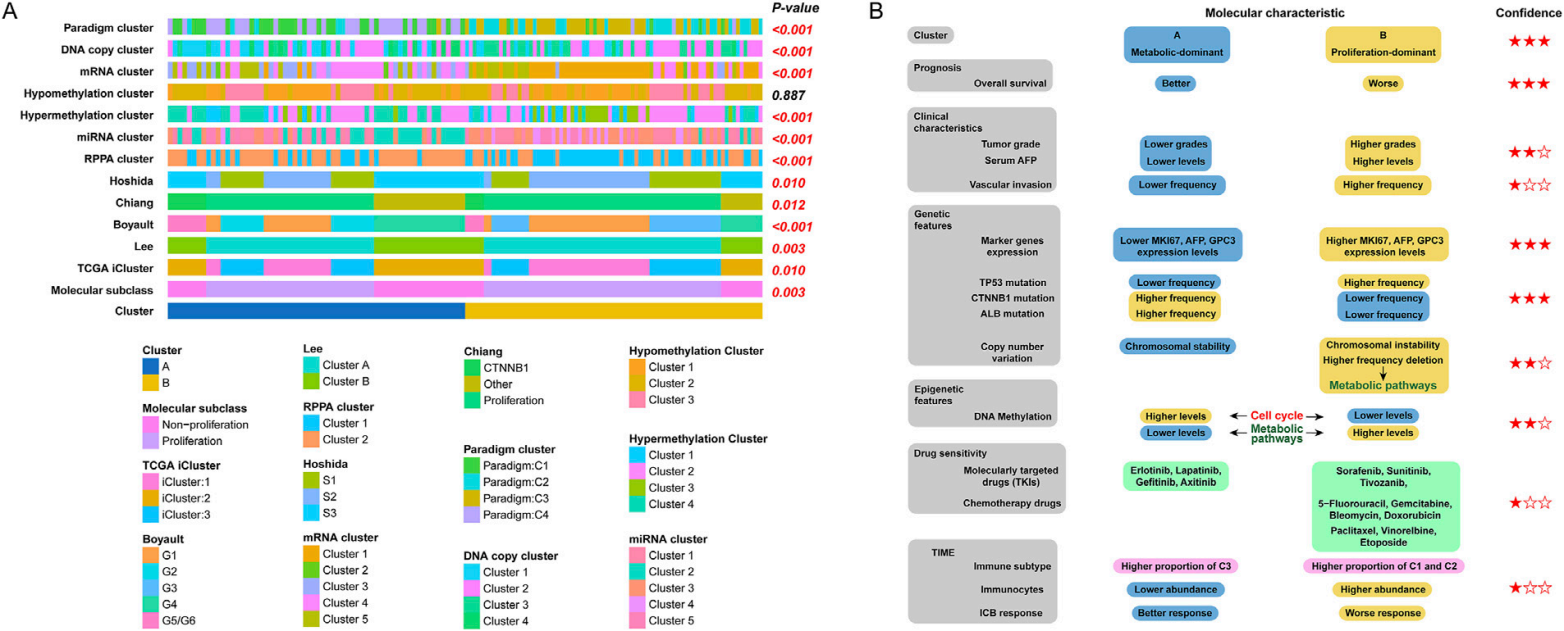
Summary of the characteristics of the two HCC subtypes. **(A)** Association between our HCC classification and molecular subtypes documented in previous studies; **(B)** Level of evidence for molecular characterization of two HCC subtypes.

To further assess the molecular characteristics of our clustering, we first compared our clustering with several previously reported HCC molecular subclasses. As shown in Figure 6A, our clustering was correlated with several previous HCC molecular subtypes, including HCC molecular subclass, TCGA icluster, Lee’s classification, Boyault’s classification, Chiang’s classification, Hoshida’s classification, RPPA cluster, miRNA cluster, hypermethylation cluster, mRNA cluster, DNA copy cluster, and paradigm cluster. In addition, our clustering was independent of the hypomethylation cluster.

In order to better visualize the differences in molecular characteristics between two HCC subclasses, we then summarised the findings of the clustering study according to the levels of evidence, which were categorized as low, medium, or high based on the type of conclusion (predictive or retrospective), significance of differences, and generalizability. The differences in molecular characteristics are summarised in Figure 6B.

### 3.6 Construction and validation of a subtype-related prognostic mRNA signature

Given that our clustering allowed for an initial assessment of a patient’s prognosis, we now intend to construct a scoring system that will allow for a more accurate and personalised prediction of each patient’s overall survival.

**FIGURE 7:**
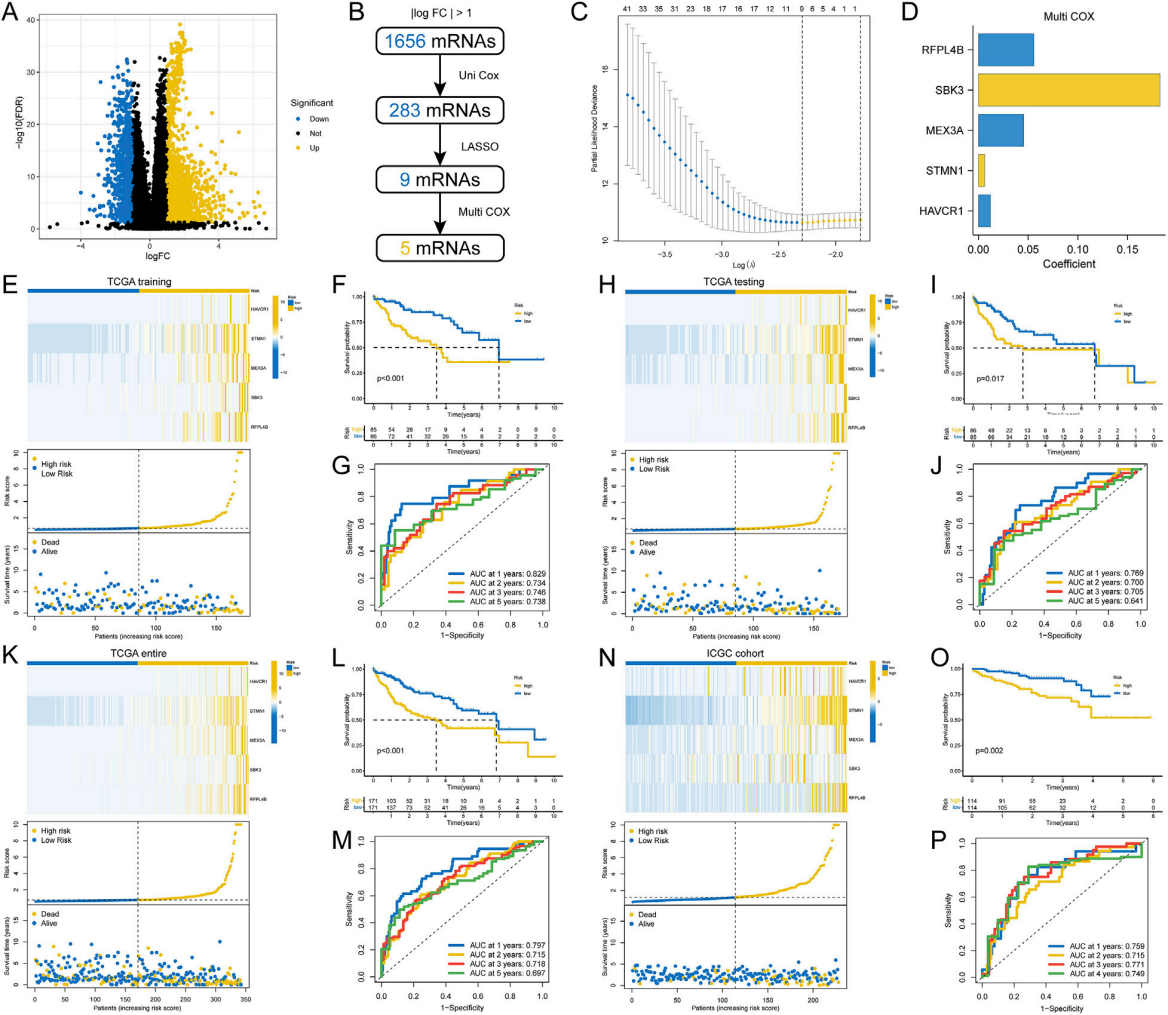
Construction and validation of a prognostic gene signature. **(A)** Volcano plot of differentially expressed genes between two HCC subtypes; **(B)** Flowchart for gene signature construction; **(C)** Cross-validation for turning parameter selection via minimum criteria in the LASSO regression model; **(D)** The coefficients of each gene screened by multivariate Cox analysis; **(E)** Heatmap of gene expression in different risk groups, distribution of riskscore, and distribution of patient mortality status; **(F)** Differences in OS for patients in different risk groups; **(G)** Time-dependent ROC curves for riskscore to predict OS; **(H–P)** Validation of the riskscore in multiple cohorts.

First, we identified a total of 1,656 DEmRNAs between the two HCC molecular subclasses using |log2 Fold Change| > 1 and *FDR* < 0.05 as the threshold. The volcano plot for differential expression analysis is shown in Figure 7A. Next, univariate Cox regression analysis was used to screen prognosis-related DEmRNAs with a threshold of *P* < 0.001. Then, LASSO and multivariate Cox regression analysis for prognosis-related DEmRNAs were performed to select the optimum prognostic mRNA signature. The flowchart of the signature construction is shown in Figure 7B. The result of LASSO regression was shown in Figure 7C and the coefficients of each variable screened by multivariate Cox analysis were shown in Figure 7D. In the TCGA training set, the heatmap and the riskplot showed higher expression of five mRNAs and more deaths in the high-risk group, respectively (Figure 7E). As expected, patients in the high-risk group had significantly worse OS than those in the low-risk group (Figure 7F). In time-dependent ROC curves, the AUC for predicting OS was 0.829 at 1 year, 0.734 at 2 years, 0.746 at 3 years, and 0.78 at 5 years (Figure 7G). Finally, we validated the signature’s performance in the testing cohort, the entire TGCA cohort, and the external validation (ICGC) cohort (Figures 7H–P). The results suggested that our signature could serve as a useful survival predictor.

### 3.7 Riskscore was correlated with clinicopathological parameters

First, we analysed the correlation between riskscore and molecular subclasses. We found that the majority of patients in the high-risk group were in cluster B and a small proportion in cluster A, while the opposite was true for the low-risk group (Supplementary Figure S4A). Furthermore, cluster B HCC did have higher riskscores than cluster A HCC (Supplementary Figure S4B). The AUC of 0.899 in the ROC curve meant that the riskscore was an accurate predictor of molecular subclasses (Supplementary Figure S4C). Then, we analysed the correlation between riskscore and two common clinial parameters, tumor stage and tumor grade. Unsurprisingly, HCC tissues with later stage or higher grade had higher riskscores (Supplementary Figures S4D, E). The above findings were also verified in the ICGC cohort (Supplementary Figures S4F–I). Finally, we analysed the correlation between riskscore and two marker genes for HCC, MKI67 and GPC3. As shown in Supplementary Figure S4J, in the TCGA cohort, the riskscore was positively correlated with MKI67 expression (r = 0.74) and GPC3 expression (r = 0.30). The results in ICGC cohort (r = 0.80, 0.28 respectively) were consistent with TCGA cohort (Supplementary Figure S4K).

### 3.8 Development and validation of a comprehensive nomogram

**FIGURE 8:**
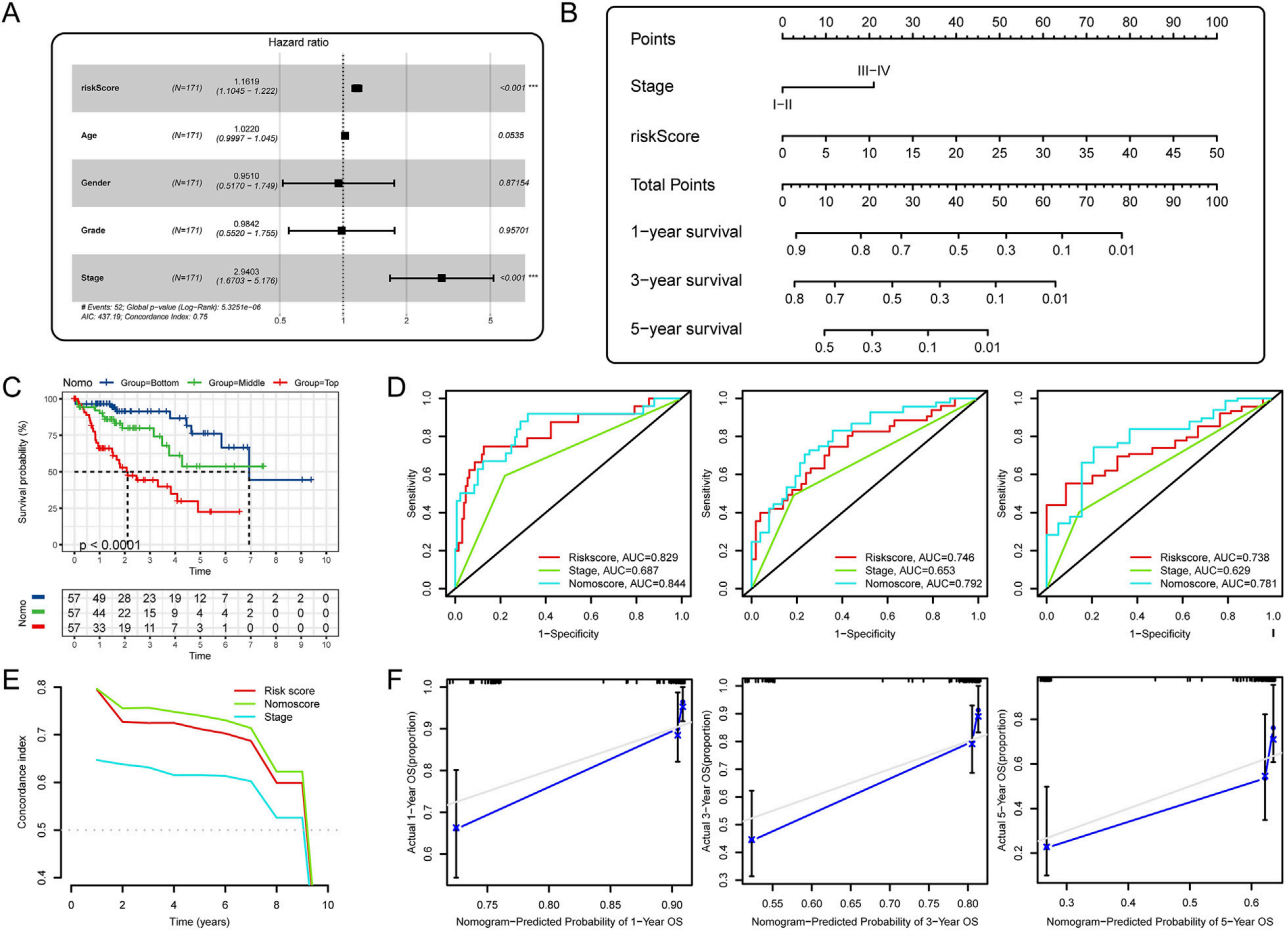
Development and validation of a comprehensive nomogram. **(A)** Multivariate Cox analysis of riskscore and clinical factors; **(B)** Development of a nomogram based on riskscore and tumor stage; **(C)** K-M analysis for OS of HCC patients stratified by nomogram points; **(D)** Time-dependent ROC curves for 1-, 3-, and 5-year OS; **(E)** C-index of prognostic indicators predicting OS; **(F)** The calibration curves for the nomogram.

To more accurately assess the prognosis of HCC patients, we plan to integrate crucial prognostic factors to construct a nomogram that allows us to predict the survival rates of HCC patients at different time points. First, we included the four most commonly used clinical characteristics (clinical grade, tumor stage, gender and age) with the riskscore in multivariate COX analysis. In the result of TCGA training cohort, riskscore and tumor stage were independent prognostic factors for OS (Figure 8A). Then, we developed a clinical nomogram based on the two variables mentioned above (Figure 8B). Using this nomogram, we were able to calculate an total point based on HCC patient’s clinical stage and riskscore to predict patient’s 1-year, 3-year, and 5-year survival rates. We trisected the patients according to their total points and compared the differences in OS between the three groups. Unsurprisingly, patients scoring in the top third had the worst prognosis, and those scoring in the bottom third had the best prognosis (Figure 8C). The results of the time-dependent ROC curves (Figure 8D) and C-index (Figure 8E) showed that the nomogram had better predictive performance than riskscore and clinical stage. The calibration curves showed the predictions were almost identical to the actual observations (Figure 8F). Finally, we verified the above conclusions in TCGA testing cohort and ICGC cohort and the results showed that the predictive performance of the nomogram was more robust than the riskscore (Supplementary Figures S5A–H).

### 3.9 Pathways associated with riskscore and therapeutic implications of riskscore in HCC

**FIGURE 9:**
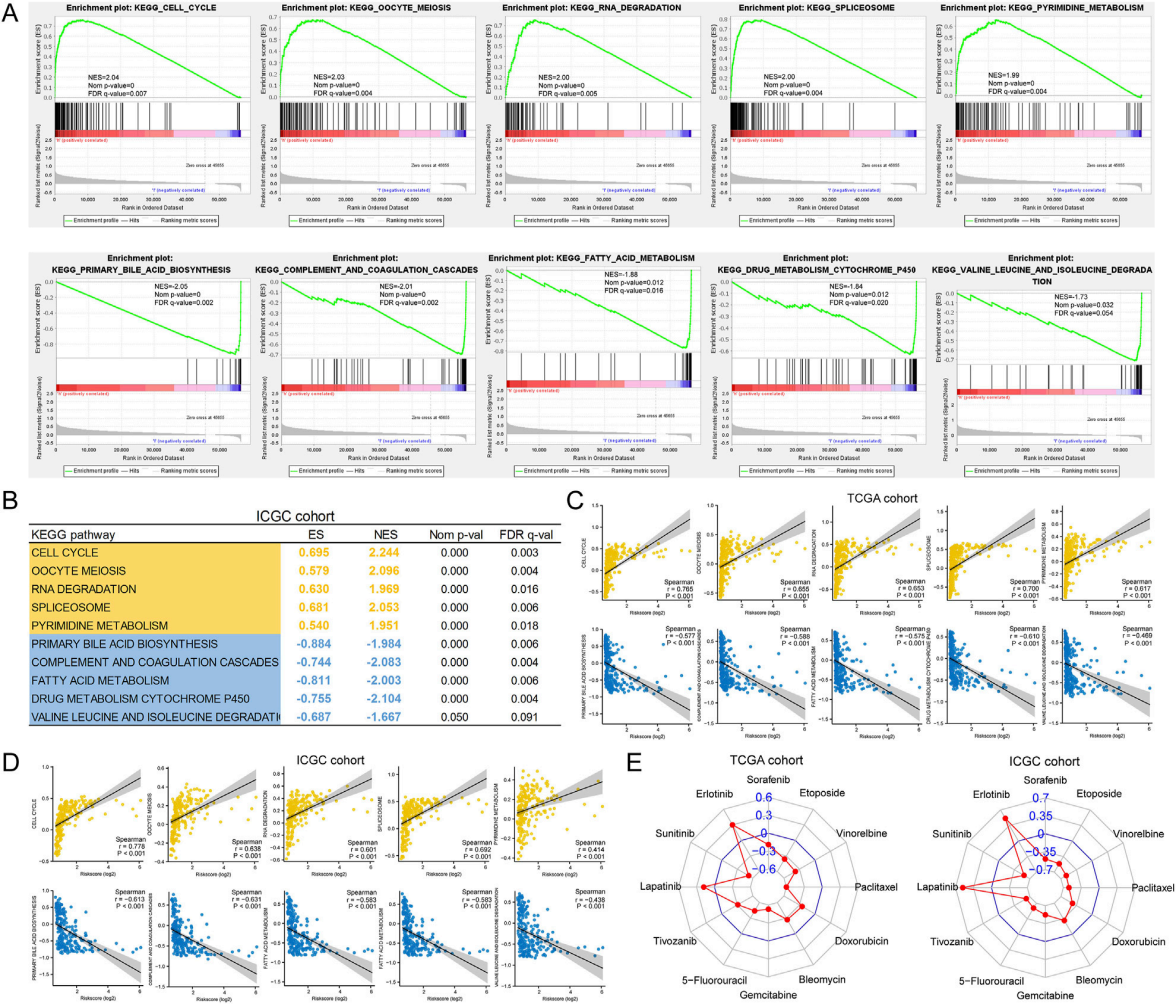
Association of riskscore with tumor heterogeneity and drug sensitivity. **(A)** Top 5 pathways correlated with riskscore screened by GSEA; **(B)** Validation of GSEA results in the ICGC cohort; **(C, D)** Correlation of riskscore with GSVA scores of pathways; **(E)** Correlation of riskscore with drug sensitivity (estimated IC50).

First, we performed GSEA to identify the KEGG pathways associated with the riskscore. In the TCGA cohort, the top 5 (ranked by NES) pathways positively correlated with riskscore were cell cycle, oocyte meiosis, RNA degradation, spliceosome and pyrimidine metabolism. The top 5 pathways negatively correlated with riskscore were primary bile acid biosynthesis, complement and coagulation cascades, fatty acid metabolism, drug metabolism cytochrome P450 and valine leucine and isoleucine degradation (Figure 9A). The enrichment results of the above 10 pathways in the ICGC cohort are shown in Figure 9B. Then, we analysed the correlation between the GSVA score for significantly enriched pathways and the riskscore. As shown in Figures 9C, D, in both cohorts, the GSVA scores for these pathways were all significantly associated with riskscore. Notably, all nine pathways, except valine leucine and isoleucine degradation, were prognosis-related pathways. This suggested that they may be the main factors through which riskscore affect patient prognosis. Finally, we assessed the value of riskscore in guiding anti-cancer drug therapy for HCC. As expected, the riskscore did correlate with sensitivity to multiple drugs (Figure 9E), and this result was consistent with previous results for our clustering.

### 3.10 Knockdown of RFPL4B represses tumor progression of Huh7 cells

**FIGURE 10:**
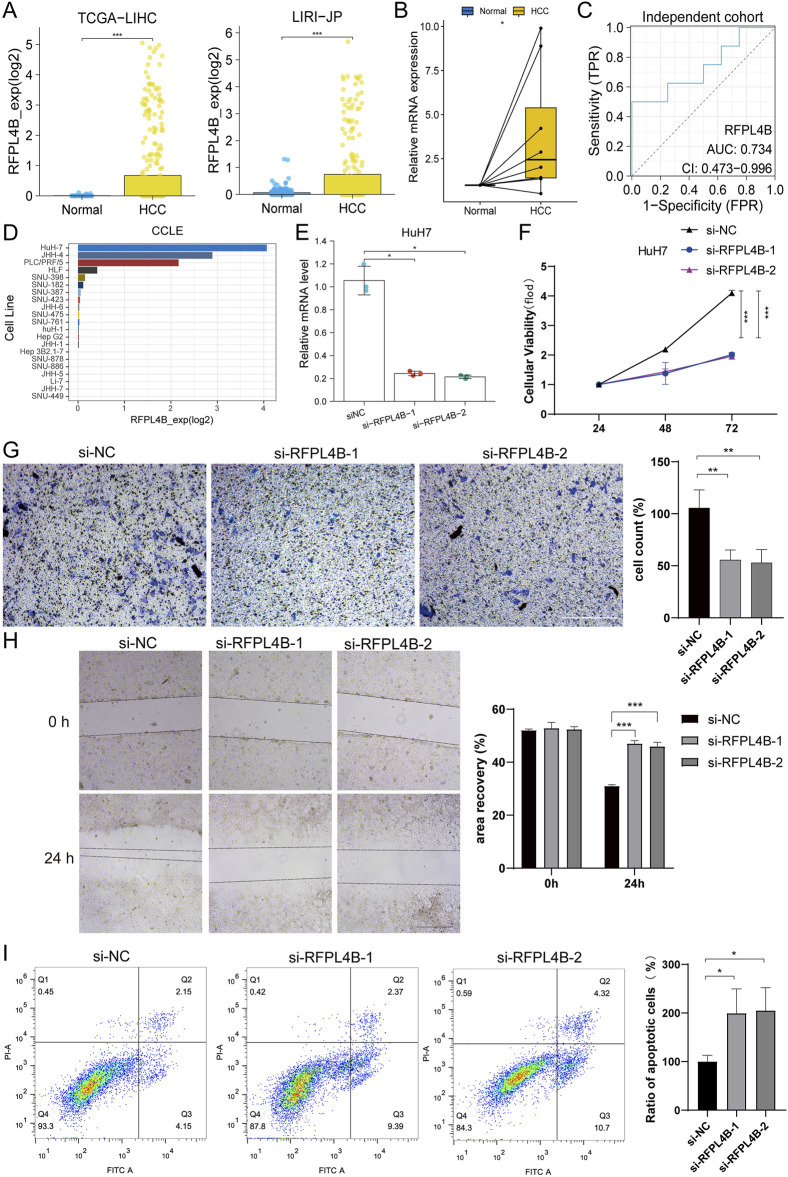
Knockdown of RFPL4B represses tumor progression of Huh7 cells. **(A–C)** Differences in the expression of RFPL4B in HCC tissues and non-cancerous tissues; **(D)** Expression levels of RFPL4B in different liver cancer cell lines in the CCLE database; **(E)** qPCR confirmed the knockdown of RFPL4B expression; **(F)** CCK-8 assay; **(G)** Transwell assay; **(H)** Wound healing assay; **(I)** Measurement of cell apoptosis by flow cytometry. (*P < 0.05, **P < 0.01, and ***P < 0.001).

Since RFPL4B has rarely been studied in cancer, we intend to further explore its biological function in HCC. First, we confirmed the up-regulation of RFPL4B in HCC tissues (Figure 10A), and this difference was also validated in an independent cohort from our center (Figures 10B, C). Next, we analyzed the expression levels of RFPL4B in different liver cancer cell lines through the CCLE database and found that RFPL4B was expressed at the highest level in the Huh7 cell line (Figure 10D). Combined with the existing conditions in our laboratory, we selected the Huh7 cell line for subsequent *in vitro* experiments. Then, we successfully knockdown the mRNA expression level of RFPL4B (Figure 10E), and the CCK-8 assay revealed that the knockdown of RFPL4B significantly inhibited cell growth (Figure 10F). Meanwhile, transwell and wound healing assays confirmed that knockdown of RFPL4B significantly inhibited the invasion and migration of Huh7 cells (Figures 10G, H). Finally, flow cytometry results showed increased cellular apoptosis rates in Huh7 cells after knocking down RFPL4B (Figure 10I).

Taken together, the above results show that knockdown of RFPL4B could repress tumor progression of Huh7 cells.

## 4 Discussion

Exposing the heterogeneity and establishing the molecular classification of cancer tissues is the key to achieving precise and individualised cancer treatment, and this has been demonstrated in a variety of cancer types, one of the best examples being breast cancer (Loibl et al., 2021). In HCC, the choice of treatment modality remains based on clinical staging, which is the basic treatment strategy for most solid tumors. The difference is that there are more treatment options available for HCC and the uniqueness of organ function has led to the inclusion of liver function in the clinical staging criteria. However, the diversity of treatment options indirectly reflects the difficulty of treatment and still does not change the fact that HCC is one of the most malignant malignancies with the highest recurrence rate and the worst prognosis (Llovet et al., 2021).

Systemic therapy is an important way to reduce tumor recurrence and prolong survival after surgery and is the main treatment for patients in the progressive stage. However, the efficacy in HCC remains unsatisfactory (Llovet et al., 2021). Some molecular features of HCC have been previously identified (Sia et al., 2017; Boyault et al., 2007; Hoshida et al., 2009; Lee et al., 2004; Chiang et al., 2008; Cancer Genome Atlas Research Network, 2017; Gao et al., 2019), but these molecular classifications are still insufficient to fully reveal the heterogeneity of tumor tissues and thus identify effective therapeutic targets, which is an important reason for the low efficiency of systemic therapy for HCC. In this study, we identified two novel molecular subtypes of HCC based on the transcriptional activity of pathways with prognostic significance. Through a comprehensive multi-omics analysis, we explored the drivers of tumor heterogeneity between subtypes and assessed the potential of this classification to guide the choice of therapeutic agents. Furthermore, a robust scoring system was also constructed to predict the overall survival of HCC patients.

As revealed by the GSVA, metabolism, cellular processes, and genetic information processing may be crucial genomic features that influence clinical outcomes in HCC patients. In cellular processes, there is no doubt that the cell cycle is one of the most critical. As a marker of malignancy, its significant activation also dominates the pivotal characteristics of proliferation HCC, and becomes a topic that has been repeatedly mentioned in recent years (Llovet et al., 2021). Metabolic reprogramming is now also considered to be a hallmark of cancer cells. Cancer cells must reprogram their metabolic state at each step of cancer progression, thereby supporting cancer growth and metastasis (Ohshima and Morii, 2021). Recent pan-cancer studies have shown that the metabolic expression characteristics of cancer are closely related to patient prognosis and sensitivity to drugs (Peng et al., 2018; Sinkala et al., 2019). In HCC, a previous study based on the non-proliferation subclass, identified two metabolic subclasses, the pericortical and the perivenous, which expressed negatively correlated gene networks (Desert et al., 2017). This demonstrated the relative independence of metabolic features from the traditional classification of HCC. As for the genetic information processing, their abnormal activation implies a poor prognosis for HCC. These pathways are mainly involved in transcriptional regulation, DNA replication, and DNA damage repair, which are inextricably linked to the occurrence and progression of cancer (Carbone et al., 2020; Jeggo et al., 2016; Gaillard et al., 2015; Modrich, 1994). It is well known that the DNA damage repair process occurs simultaneously with cell cycle arrest. However, we found that the transcriptional expression characteristics of both seem to be positively correlated in HCC. Limited by how GSVA works, it is unclear what cause-and-effect relationship is hidden behind this, and whether this pattern also exists in other cancer types or normal tissue cells. Perhaps, it is necessary to conduct an in-depth experimental exploration based on this issue in the future. However, it is undeniable that two novel HCC subtypes were identified. One subtype has a transcriptional pattern close to that of normal hepatocytes, and a higher metabolic pathway activity dominates a better prognosis. The other subtype was characterized by significant enrichment in DNA damage repair and cell cycle, and low activity of many metabolic pathways, resulting in higher heterogeneity and poorer survival.

The genomic features of these two subclasses also provide clues to their heterogeneity. It is currently believed that mutated TP53 loses its original regulatory functions, which are mainly focused on cell cycle and DNA repair (Vaddavalli and Schumacher, 2022). Activation of the Wnt/β-catenin pathway by mutant CTNNB1 plays a crucial role in regulating liver metabolism (Zucman-Rossi et al., 2015; Rebouissou and Nault, 2020). This type of HCC has a unique type of metabolic pattern (Senni et al., 2019; Rebouissou et al., 2016; Calderaro et al., 2017). Therefore, TP53 mutations and CTNNB1 mutations are essential drivers of HCC heterogeneity. In addition, we identified a higher frequency of ALB mutations in cluster A. Although the biological significance of this alteration is not yet clear, previous studies have speculated its close association with metabolic reprogramming in the progression of HCC (Cancer Genome Atlas Research Network, 2017). Chromosome stability is also a significant difference between proliferation class and non-proliferation class (Llovet et al., 2021). As expected, we found that cluster B had a higher frequency of CNV. More importantly, through further rigorous screening, we found that deletion of metabolism-related genes leading to down-regulation of their transcript levels was another driving factor for the absence of metabolic activity in cluster B HCC. The third driver of HCC heterogeneity in our study originated from the analysis of epigenetic data. The methylation levels of genes related to cell cycle and metabolism also indirectly regulate the transcriptional profile of HCC.

Over 90% of HCC cases occur in the setting of chronic liver disease. Cirrhosis from any aetiology is the most decisive risk factor for HCC (Marrero et al., 2018; European Association for the Study of the Liver, 2018). Major risk factors for HCC include chronic alcohol consumption, diabetes or obesity-related NASH, and infection with HBV or HCV. The proliferation subclass is more common in HBV-related HCC, whereas non-proliferation subclass is more prevalent in alcohol-related HCC and HCV-associated HCC (Llovet et al., 2021). However, the two HCC subclasses we identified do not appear to differ significantly in aetiology, at least not in relation to HBV and HCV infection and alcohol consumption. This begs the question of whether there are other etiologies driving this heterogeneity in HCC. Unfortunately, there is a lack of etiologic data for in-depth study. As for clinicopathological parameters, we found that cluster B HCC was more poorly differentiated, with patients having higher serum AFP levels and a higher frequency of vascular invasion. This is similar to the traditional HCC classification (Llovet et al., 2021). We also observed higher mRNA and protein expression levels of ki67 and GPC3 in cluster B, which again demonstrates the distinct malignant features of this HCC subtype.

It can be seen that our classifier is both consistent and different from the multiple HCC classification criteria that have been established previously. This suggests that the molecular features of HCC are initially understood, but there are still potential drivers of heterogeneity that remain unexplored. Fortunately, we found that the inherent heterogeneity of HCC can be a potential target for treatment. For example, cluster B HCC was more sensitive to sorafenib, 5-FU, paclitaxel and doxorubicin, as we predicted. These drugs are also the current clinical options for the treatment of HCC. However, because sensitivity data for some newly approved first-line therapeutic agents are not yet available, such as lenvatinib, bevacizumab, etc., we could not assess the response of different subclasses of HCC to these agents. Therefore, in the future, it will be necessary to routinely apply high-throughput techniques in clinical studies to validate the value of our classifier for the treatment of HCC. In addition, to further improve the clinical value of our classifier, we developed a robust risk scoring system consisting of weighted expression levels of 5 genes (RFPL4B, SBK3, MEX3A, STMN1, and HAVCR1). With this system, we can predict the overall survival of patients, determine the molecular subtype of HCC tissues, and predict the response of tissues to drug therapy. Moreover, the combination of risk score and clinical stage further improves the predictive accuracy of patient prognosis.

The results of the enrichment analysis showed that the riskscore was closely related to the differential pathways between the two HCC subclasses we identified. Therefore, it can be inferred that the above five genes may play crucial roles in HCC. Previous studies have shown that MEX3A and HAVCR1 were associated with hypoxia and immunity and may serve as potential prognostic markers for HCC (Ding et al., 2022; Hu et al., 2020; Wang et al., 2022) More importantly, the expression of HAVCR1, a gene related to fatty acid metabolism, can promote the proliferation, motility, and invasion of HCC cells (Zhu et al., 2022), which is consistent with the findings of our study. As for STMN1, as an oncogene, its role in HCC has been progressively confirmed. Its encoded protein, stathmin 1, was found to be upregulated in HCC and associated with cancer cell proliferation (Li et al., 2005), polyploidy and tumor-cell invasion (Hsieh et al., 2010). STMN1 expression was positively correlated with the expression of cell cycle-related genes, and high STMN1 expression also implied that HCC tissues are more poorly differentiated and patients are prone to early recurrence and microvascular invasion (Hsieh et al., 2010; Wang and Yang, 2021; Xiao et al., 2022; Cai et al., 2022). In addition, the role of STMN1 in DNA damage repair has also been shown to be prognostically relevant (Huo et al., 2022). Since STMN1 plays a critical role in HCC, more and more studies focusing on the molecular mechanisms behind it have been conducted. Currently, miR-223, miR-101, KPNA2 and E2F1 have been identified as regulators of STMN1 in HCC (Wong et al., 2008; Wang et al., 2014; Xu et al., 2013; Zheng et al., 2015; Drucker et al., 2019; Chen et al., 2013). On the therapeutic side, STMN1 has also demonstrated potential as an effective therapeutic target (Wang and Yang, 2021; Xu et al., 2013; Zheng et al., 2015; Chen et al., 2013; Wang et al., 2009; Zhou et al., 2011; Ghasemi et al., 2013; Tseng et al., 2016). Consistent with our prediction, gene silencing of STMN1 by RNAi showed a distinct synergistic effect in the combined treatment with nab-paclitaxel (Zhou et al., 2011). In addition, in breast cancer cells, stathmin is overexpressed in the presence of p53 mutation, and wild-type p53 can repress stathmin transcription. Moreover, the inhibition of stathmin significantly reduces the proliferation, viability, and clonogenicity of mutant p53 cells, restores cell cycle regulation, activates apoptosis, and recovers specific wild-type functions in cancer cells harboring mutant p53 (Alli et al., 2007). Thus, there has been substantial evidence to support our findings, which leads us to believe strongly that STMN1 may be another key driver of HCC heterogeneity. As for RFPL4B and SBK3, their roles in HCC are not yet clear. In our study, we confirmed by *in vitro* experiments that the knockdown of RFPL4B could repress tumor progression of Huh7 cells. There is no doubt that comprehensive studies of these five genes will significantly enhance our understanding of HCC.

However, it is essential to note that this study has the following limitations. First, this study only included two HCC cohorts with a limited sample size. There is a need to expand the sample size for further validation of the study’s findings, and the clinical implications of this study need to be evaluated in practice. Next, results such as immune cell abundance and drug sensitivity are based only on algorithmic predictions, which have a low level of evidence and require validation by *in vivo* and *in vitro* experiments. Then, this study dealt only with the transcriptional level. Differences in heterogeneity should be subsequently assessed at the protein level. Finally, the effect of RFPL4B on HCC needs to be confirmed in more types of HCC cell lines or even in animal experiments.

## 5 Conclusion

We identified two heterogeneous subtypes of HCC, the metabolism-dominant and proliferation-dominant types. These two HCC subtypes have different clinical features, genomic drivers, therapeutic susceptibility and immune microenvironment. Meanwhile, we constructed a gene signature to predict molecular subtypes, overall survival, and drug sensitivity. Our findings are beneficial in exposing the causes of HCC heterogeneity and exploring new therapeutic targets.

## Data Availability

The datasets presented in this study were obtained from publicly available databases. The raw data can be found here:TCGA cohort (https://portal.gdc.cancer.gov/). ICGC cohort (https://dcc.icgc.org/).

## Supplementary material

SUPPLEMENTARY FIGURE S1 Differences in transcriptional profile between cluster A HCC, cluster B HCC and noncancerous liver.

SUPPLEMENTARY FIGURE S2 Differences in the clinical characteristics of patients with different HCC subtypes.

SUPPLEMENTARY FIGURE S3 Differences in the mutation profiles of different HCC subtypes. **(A)** Mutation profiles of the top 15 genes with the highest mutation rates in the TCGA cohort between the two HCC subtypes; **(B)** Differences in TMB between two HCC subtypes in the TCGA cohort.

SUPPLEMENTARY FIGURE S4 Correlation of riskscore with clinical parameters. **(A, B)** Correlation of riskscore with HCC molecular subtypes; **(C)** ROC curves for predicting HCC molecular subtypes by riskscore; **(D)** Differences in riskscore in tissues with different levels of differentiation; **(E)** Differences in riskscore in tissues with different stages; **(F–I)** Validation of the above results in the ICGC cohort; **(J, K)** Correlation of riskscore with MKI67 and GPC3 expression. (***P* < 0.01, and ****P* < 0.001).

SUPPLEMENTARY FIGURE S5 Validation of the nomogram. **(A–D)** TCGA testing cohort; **(E–H)** ICGC cohort.

SUPPLEMENTARY TABLE S1 The sequences of siRNA.

SUPPLEMENTARY TABLE S2 The sequences of qPCR primers.

SUPPLEMENTARY TABLE S3 41 prognosis-related pathways.
