# Supplementary figures and images for: Identification of two heterogeneous subtypes of hepatocellular carcinoma with distinct pathway activities and clinical outcomes based on gene set variation analysis
Source: Front Genet. 2024 Sep 10;15:1441189. doi: 10.3389/fgene.2024.1441189 (PMC11423295; doi:10.3389/fgene.2024.1441189)

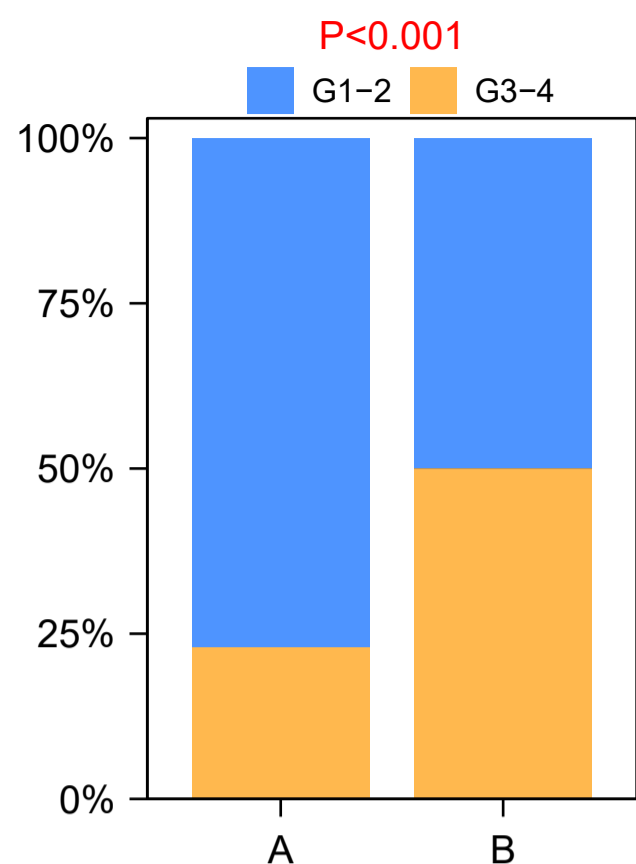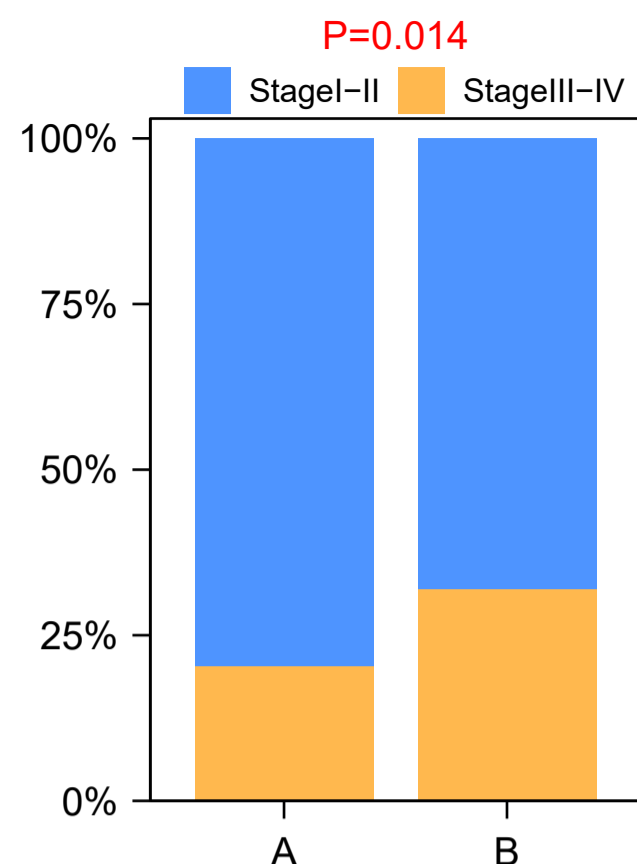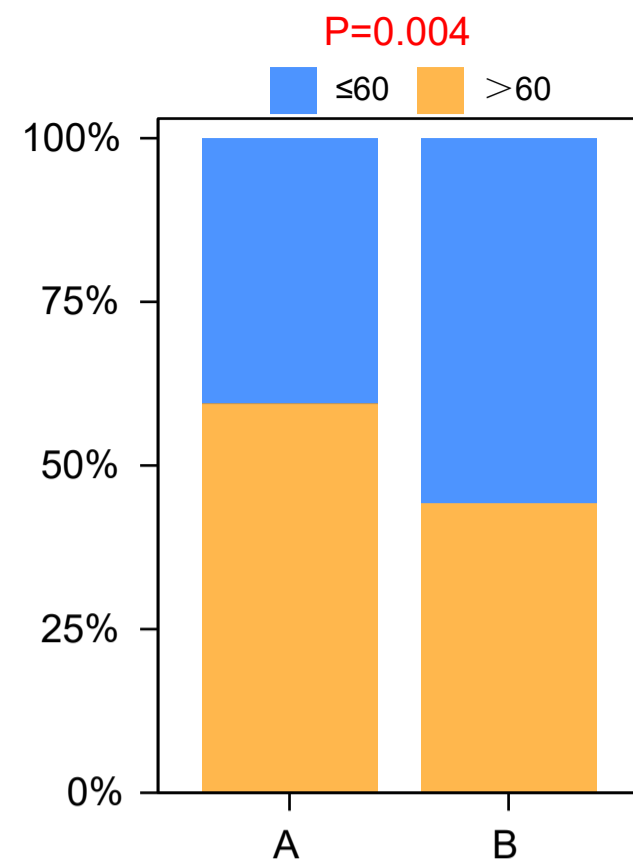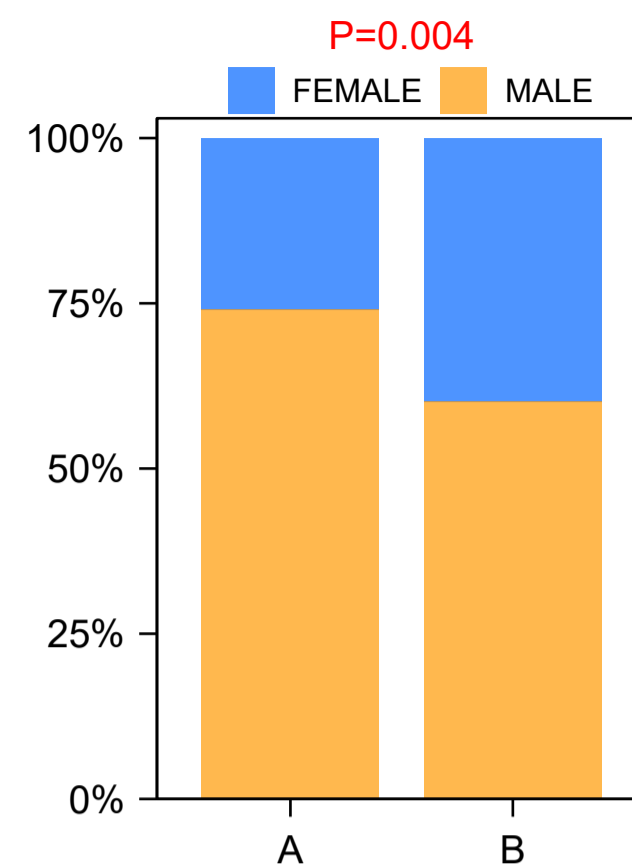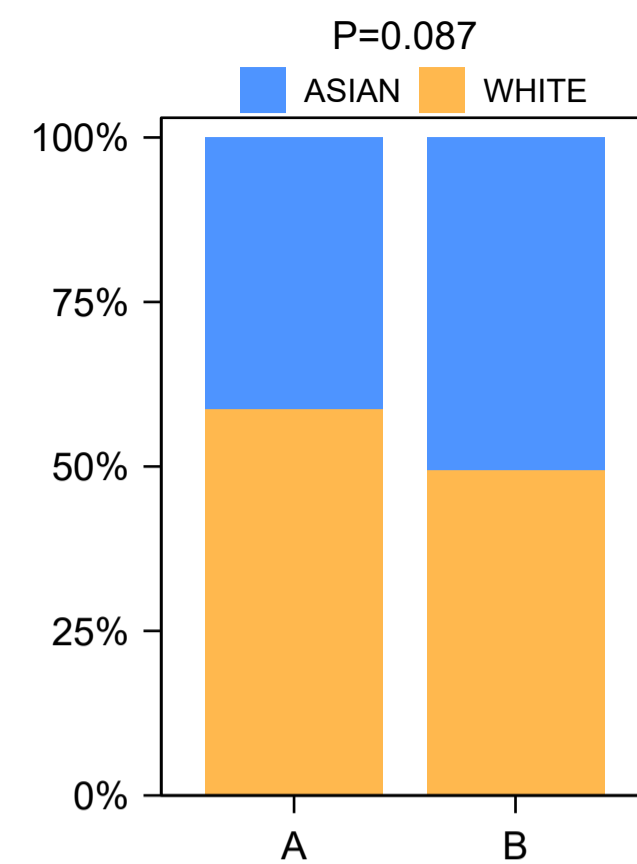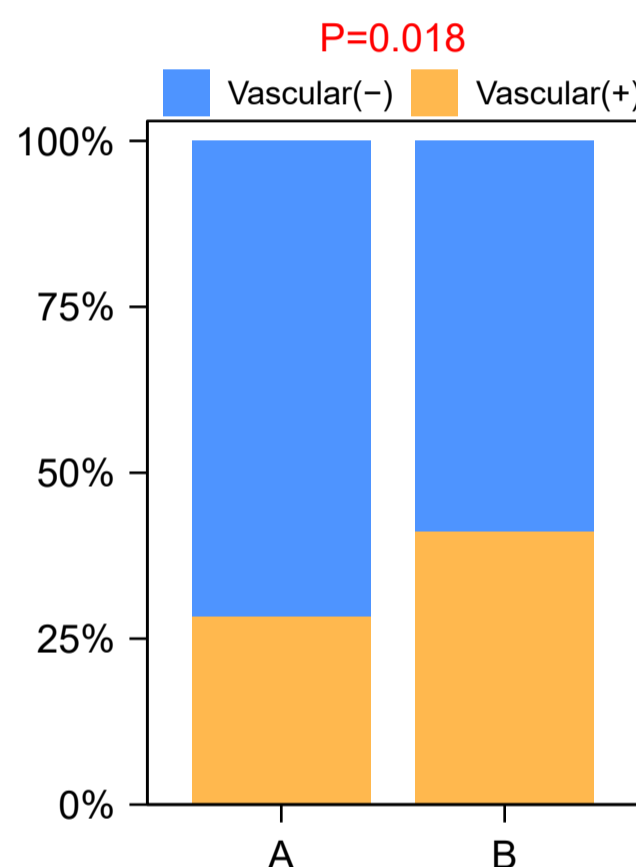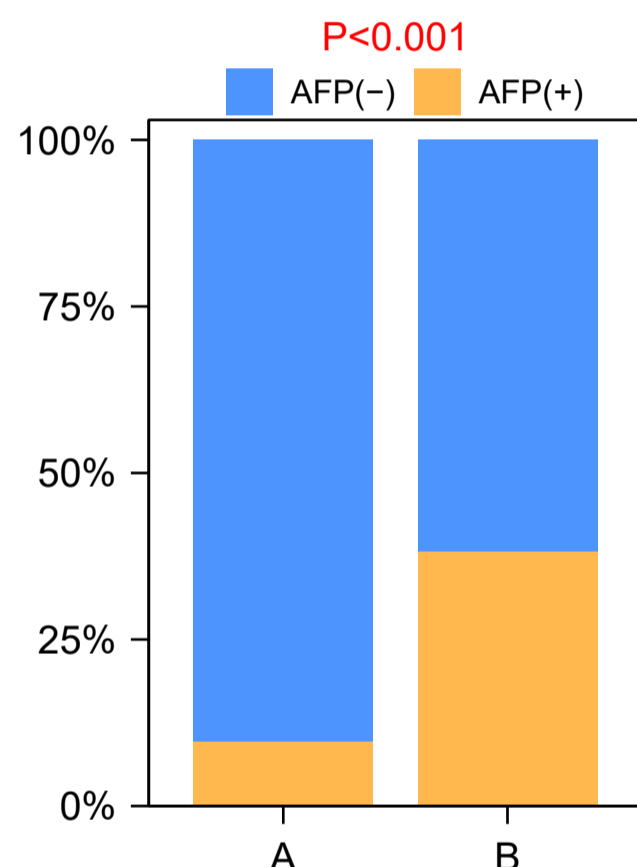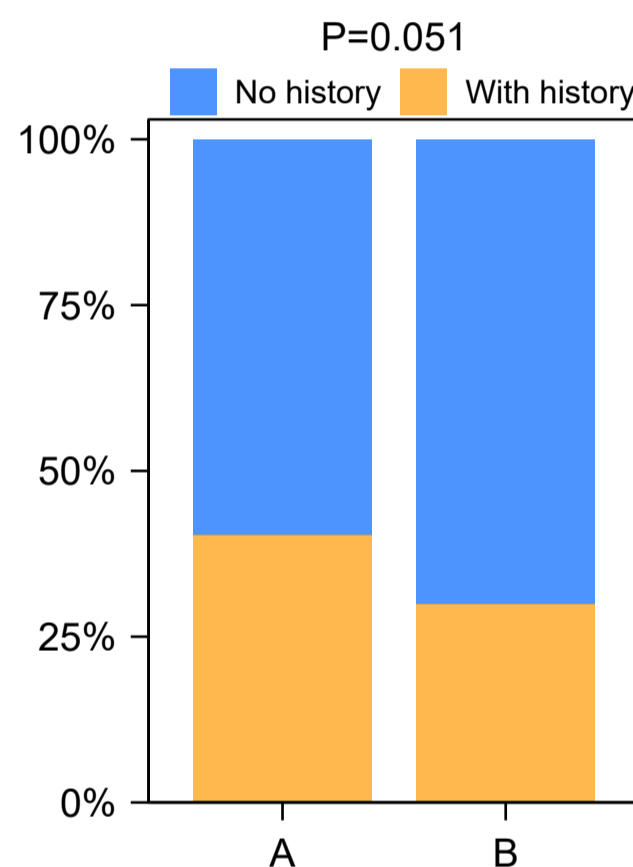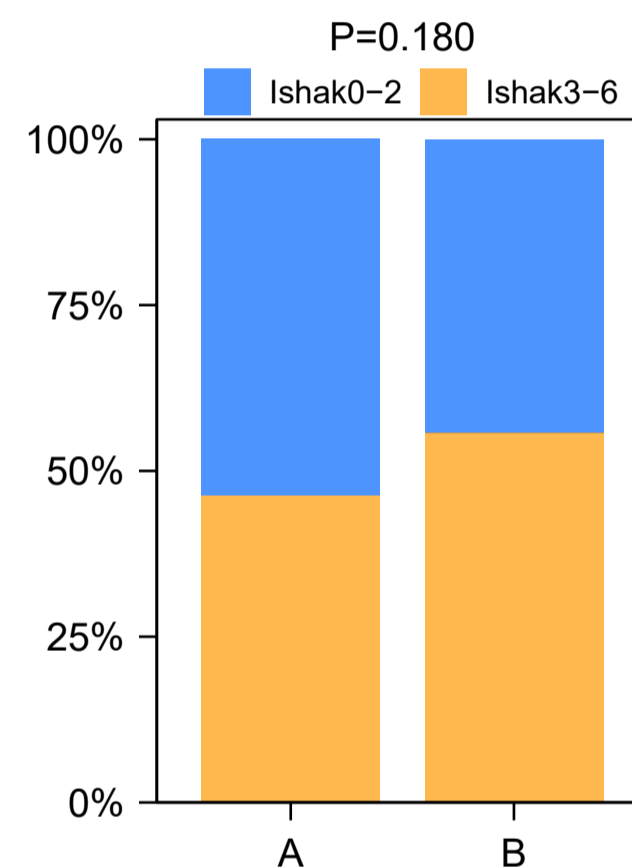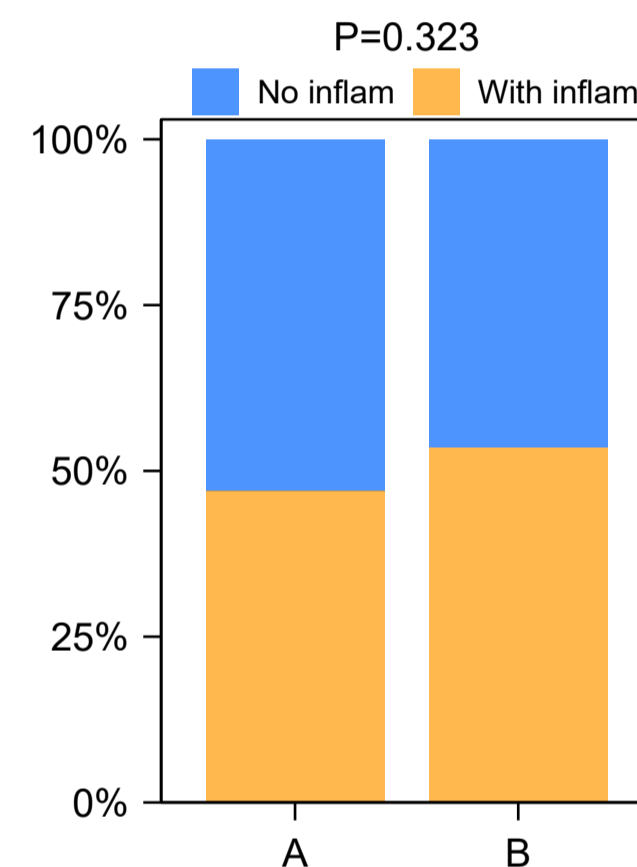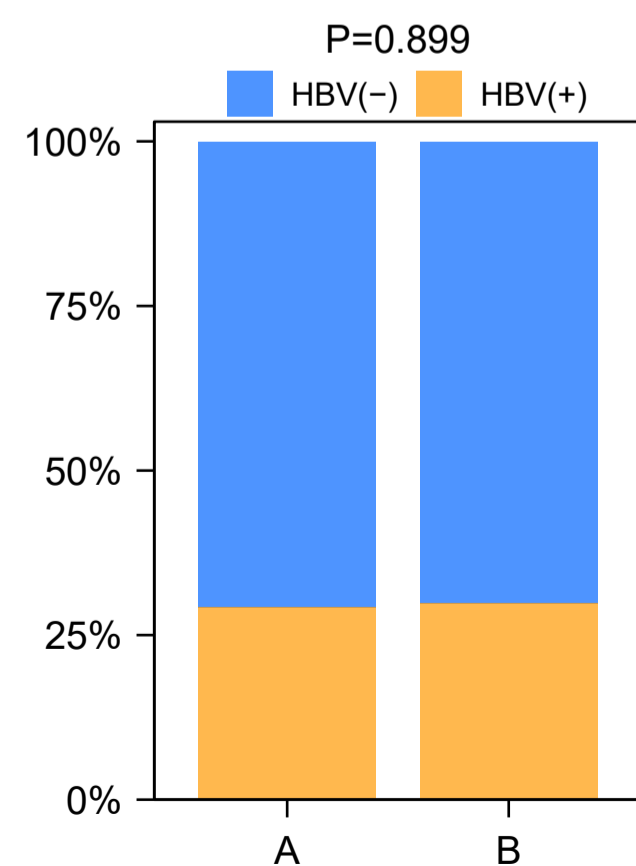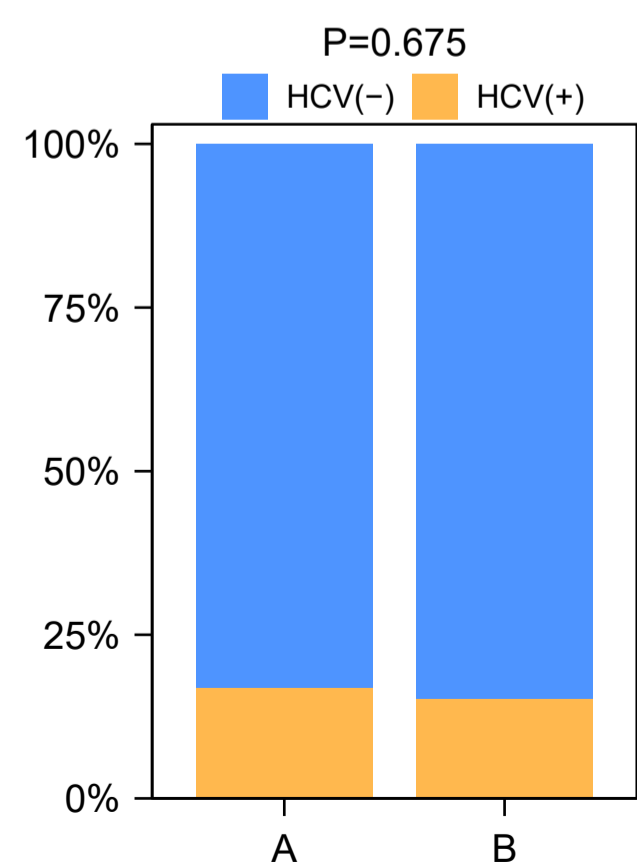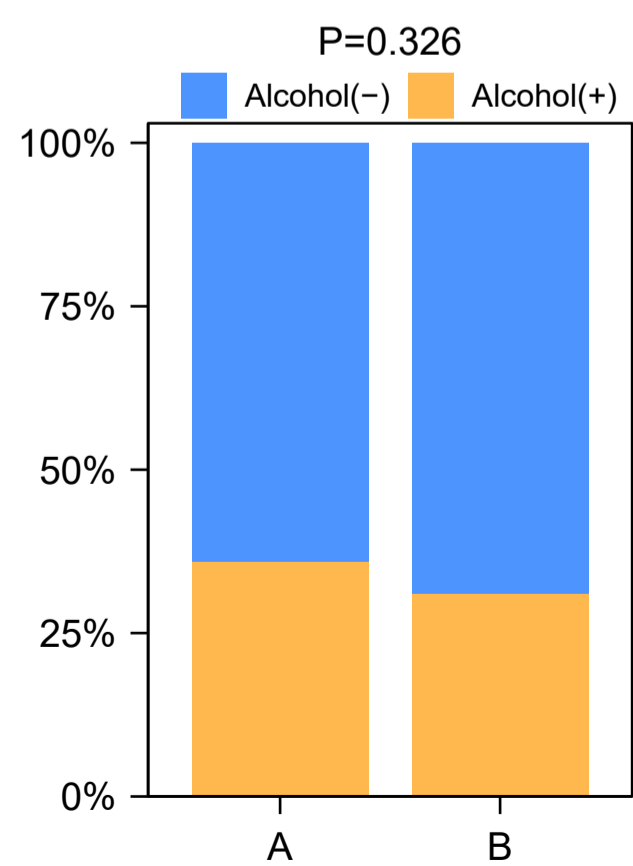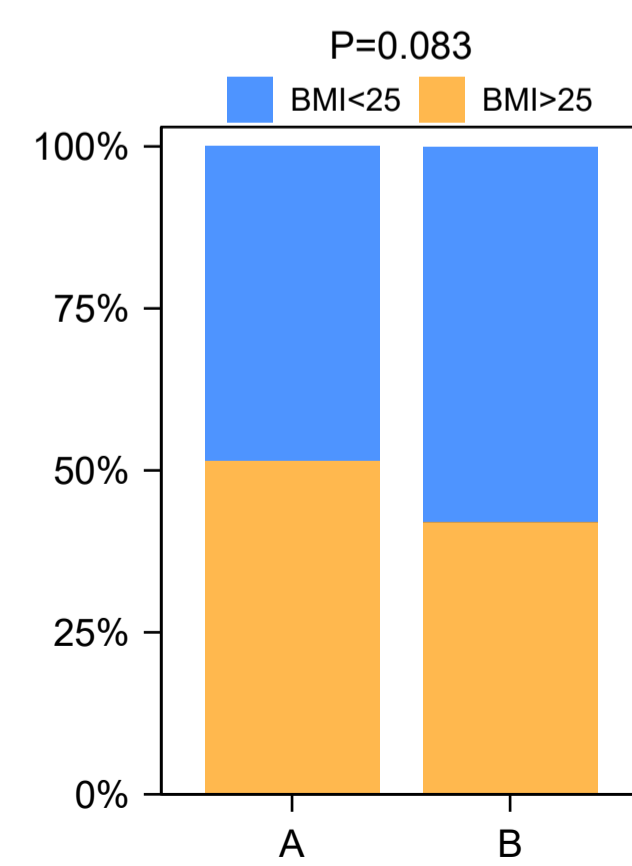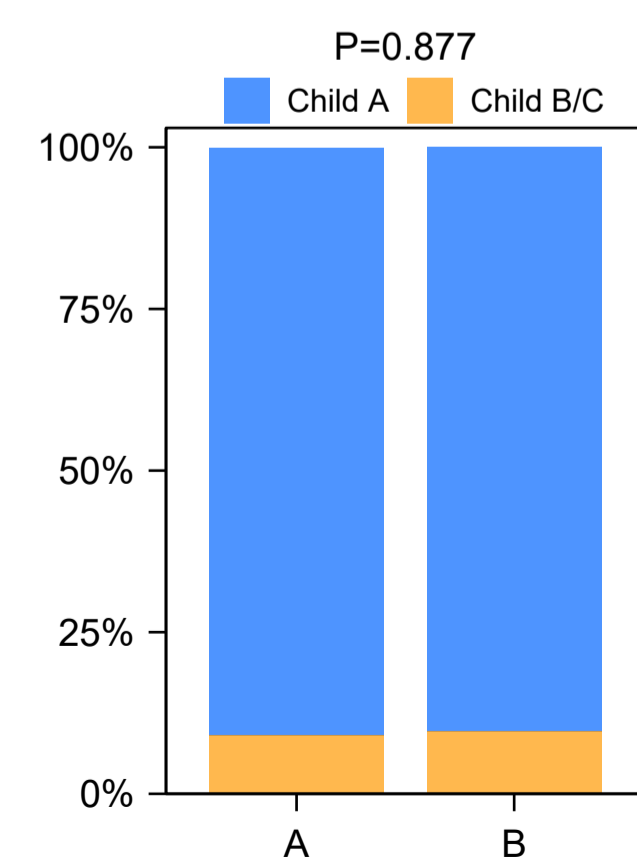

Supplement: Supplementary file 1 [file DataSheet2.PDF]

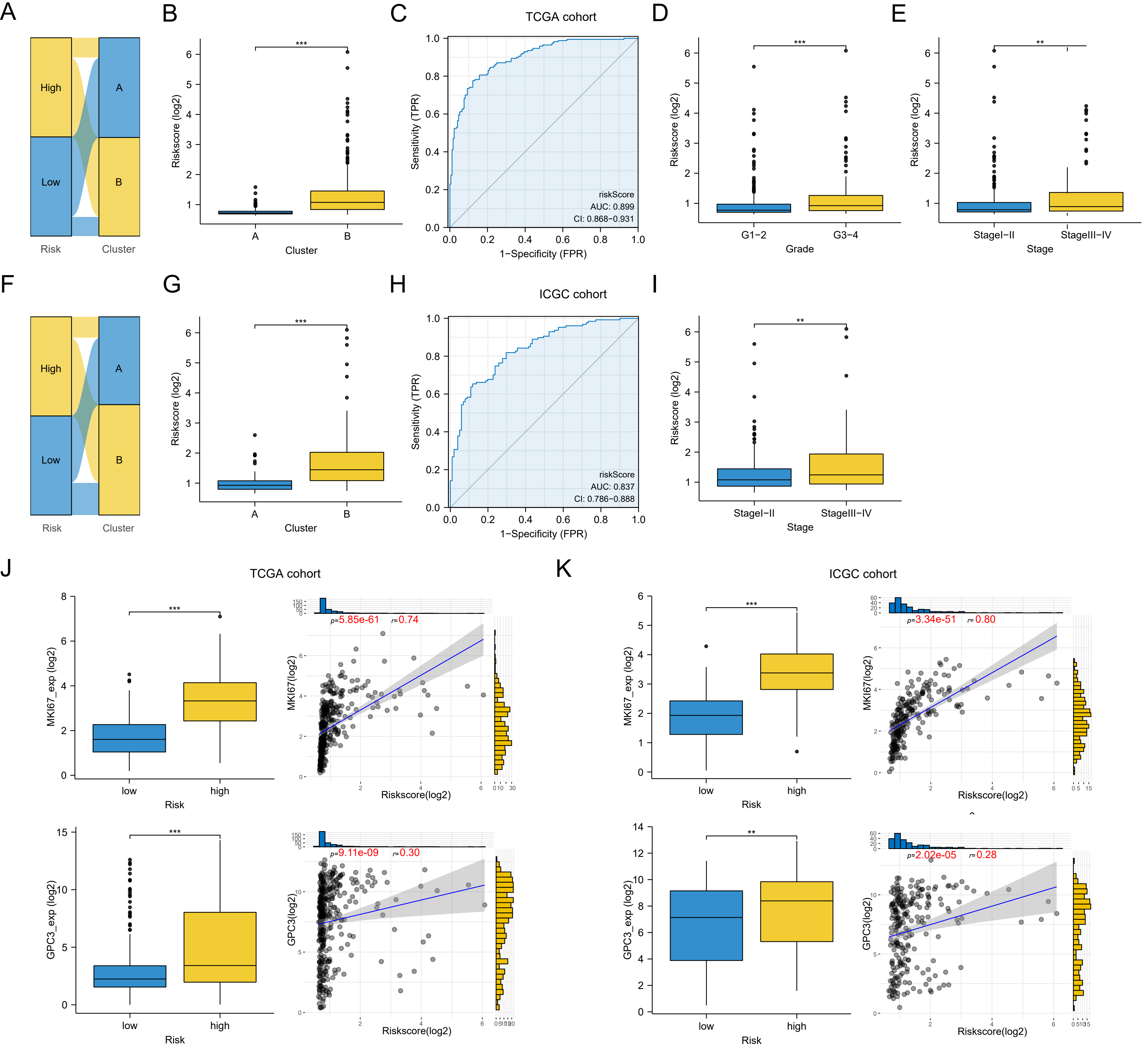

Supplement: Supplementary file 3 [file DataSheet4.PDF]

A

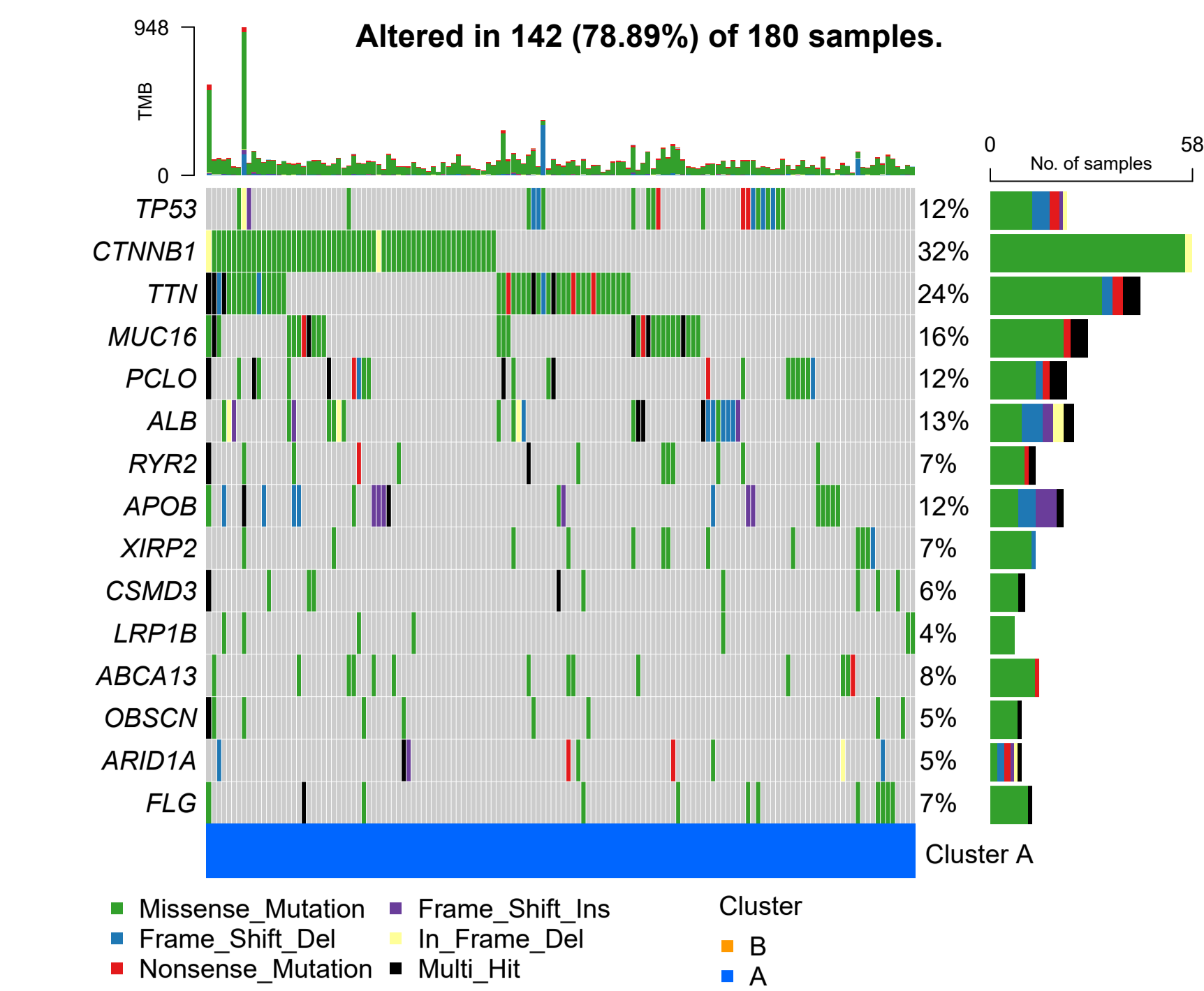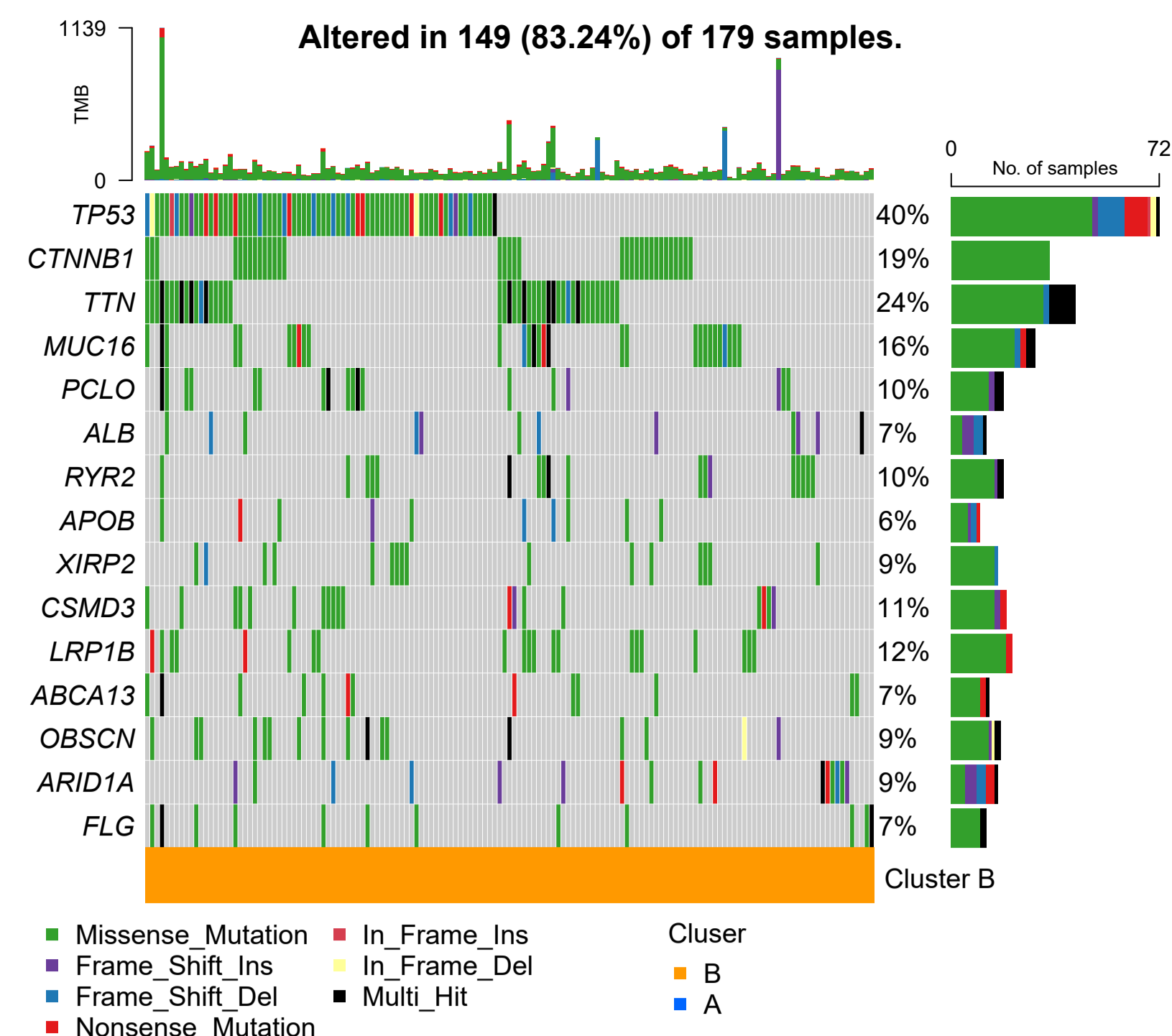

B

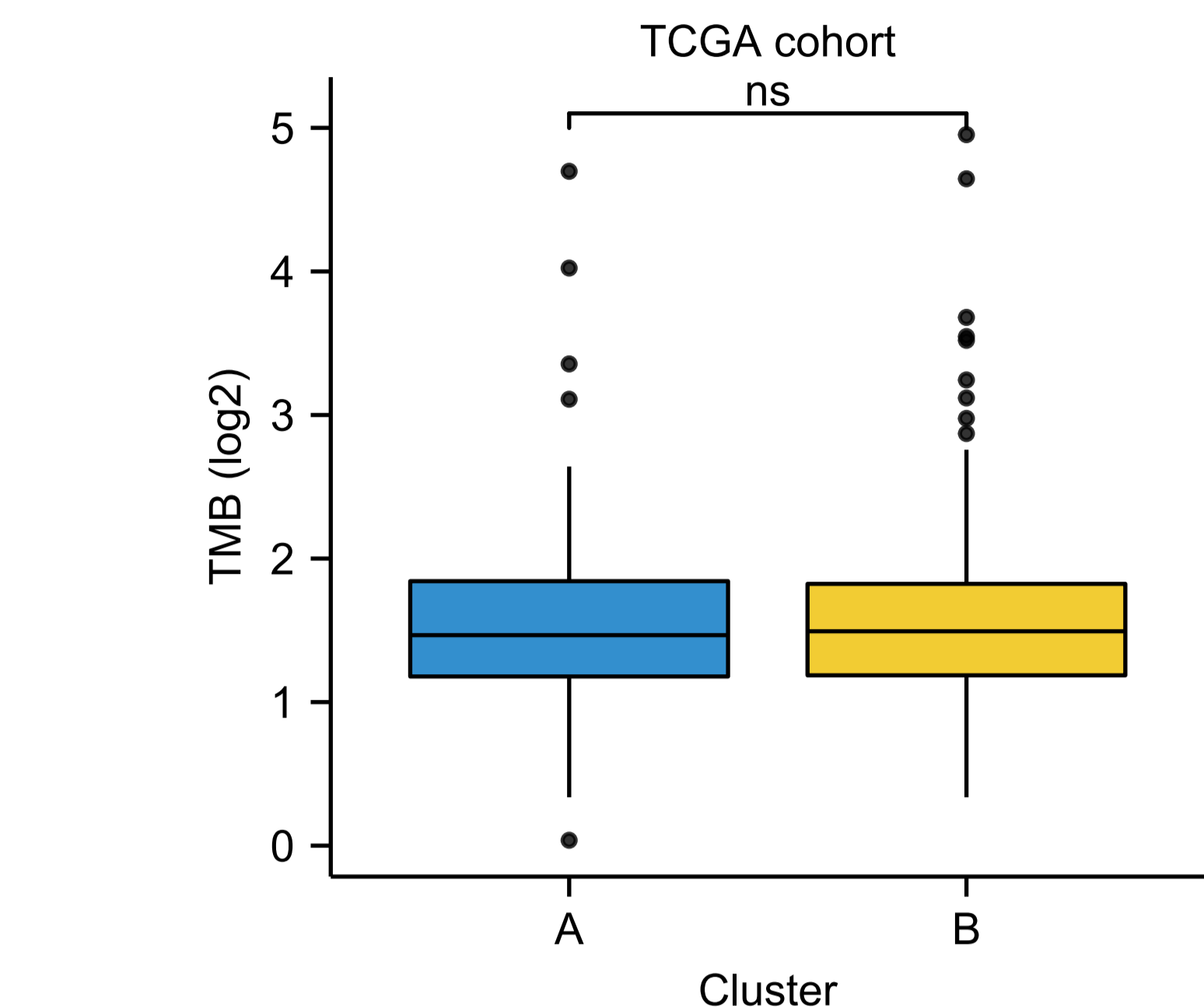

Supplement: Supplementary file 5 [file DataSheet3.PDF]

TCGA

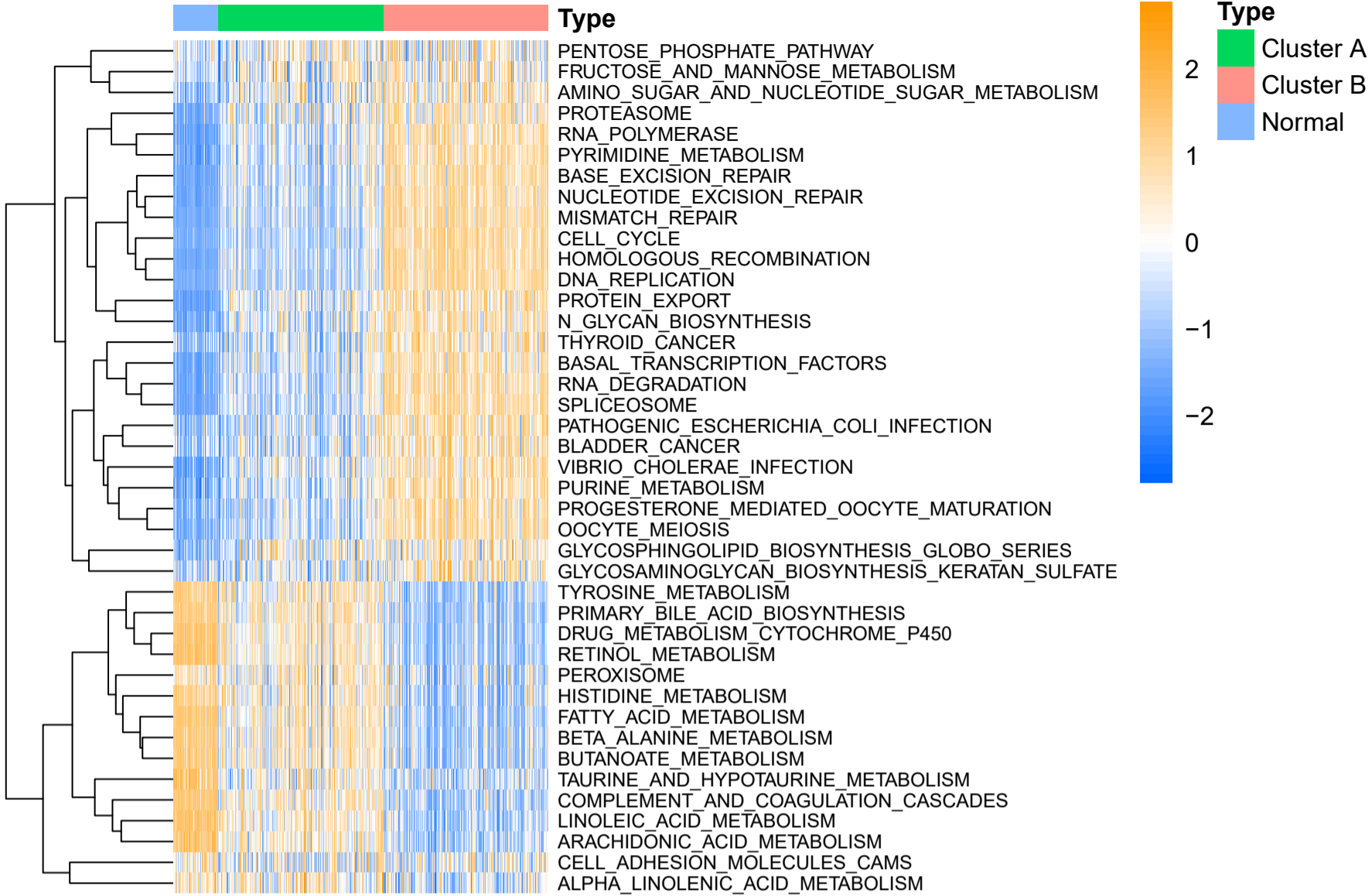

ICGC

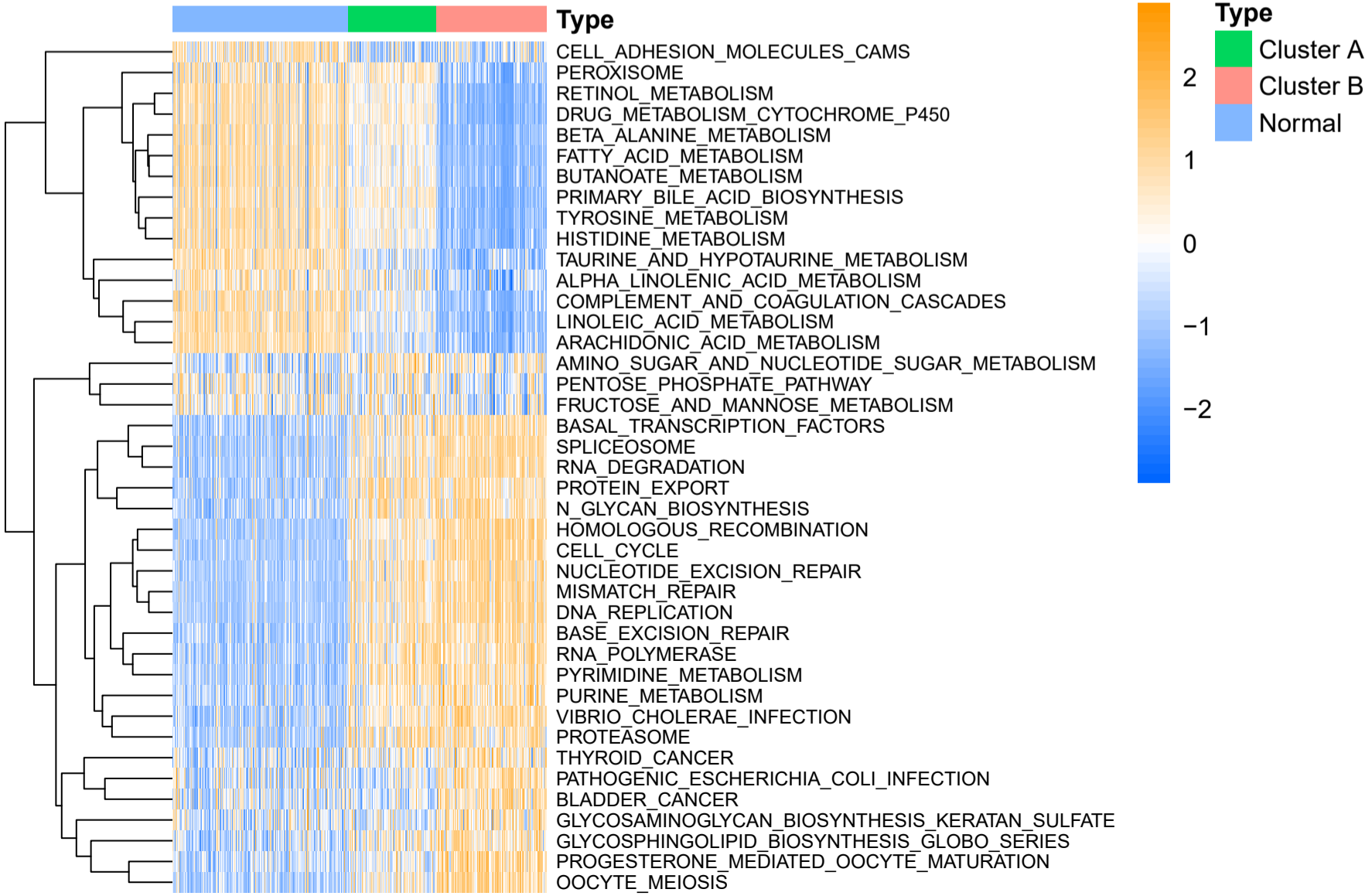

Supplement: Supplementary file 6 [file DataSheet1.PDF]

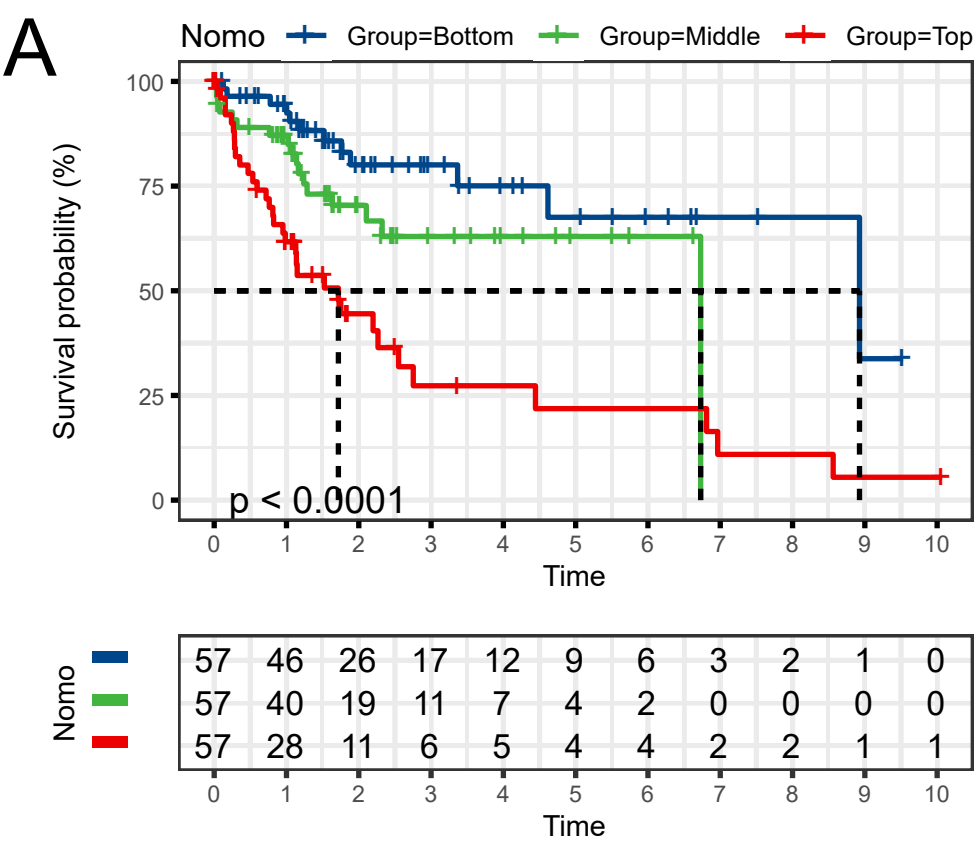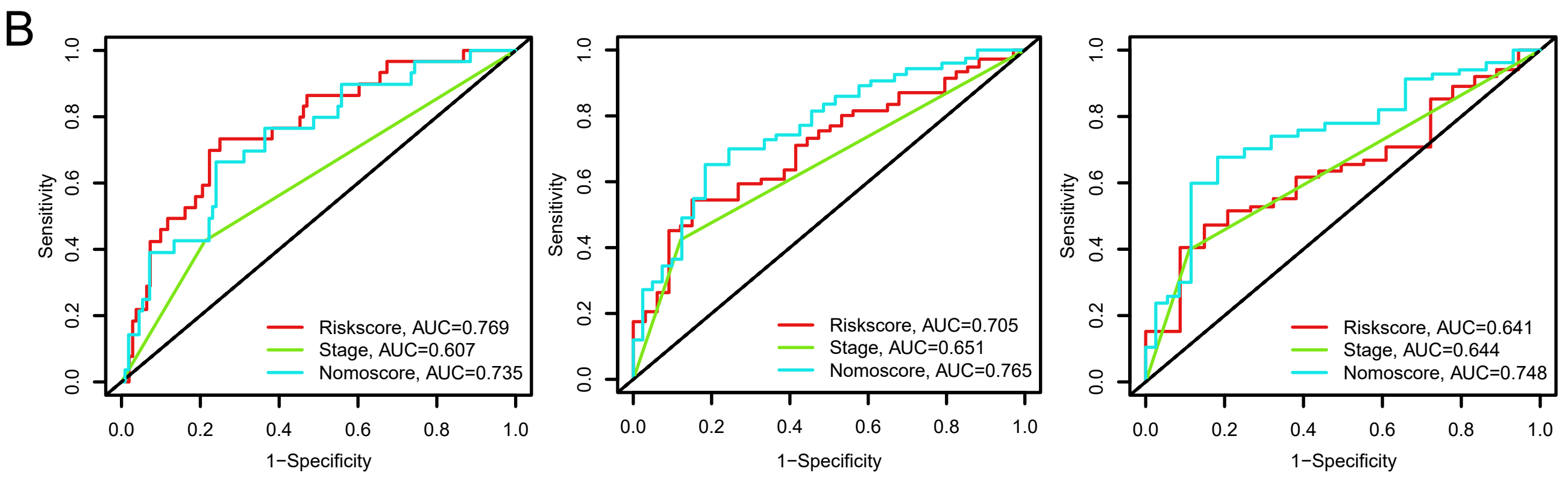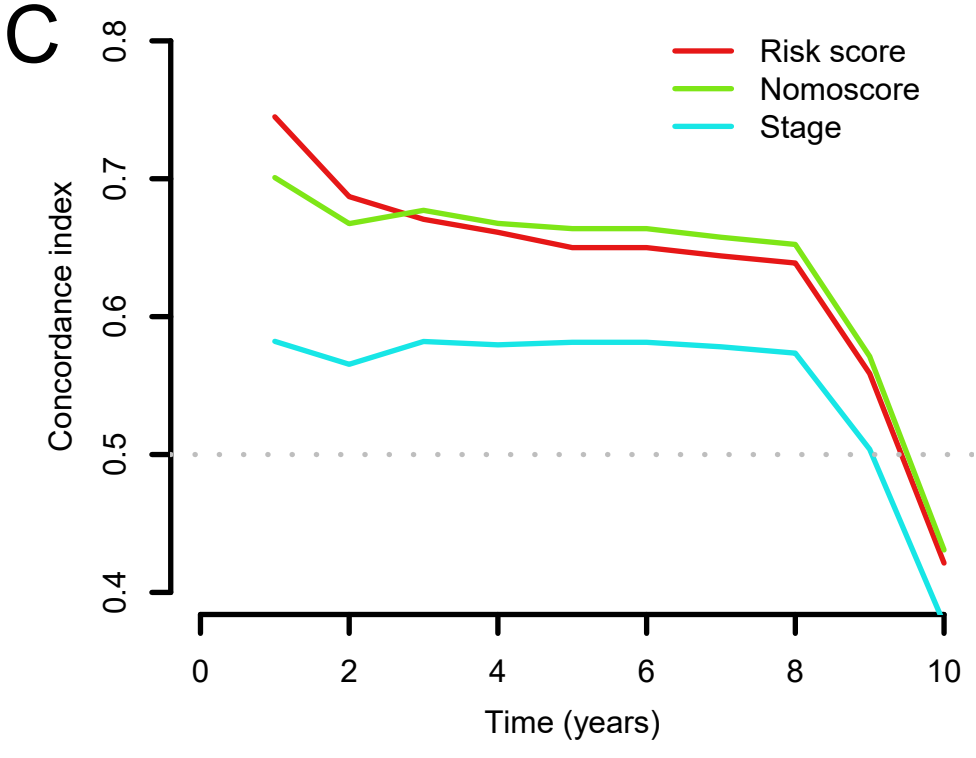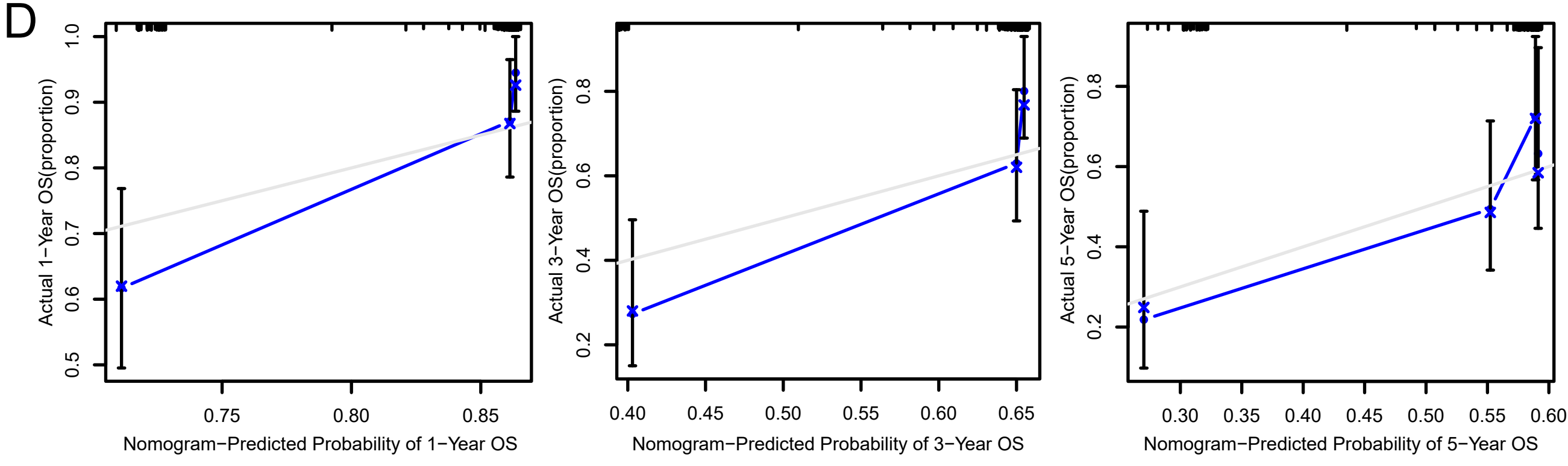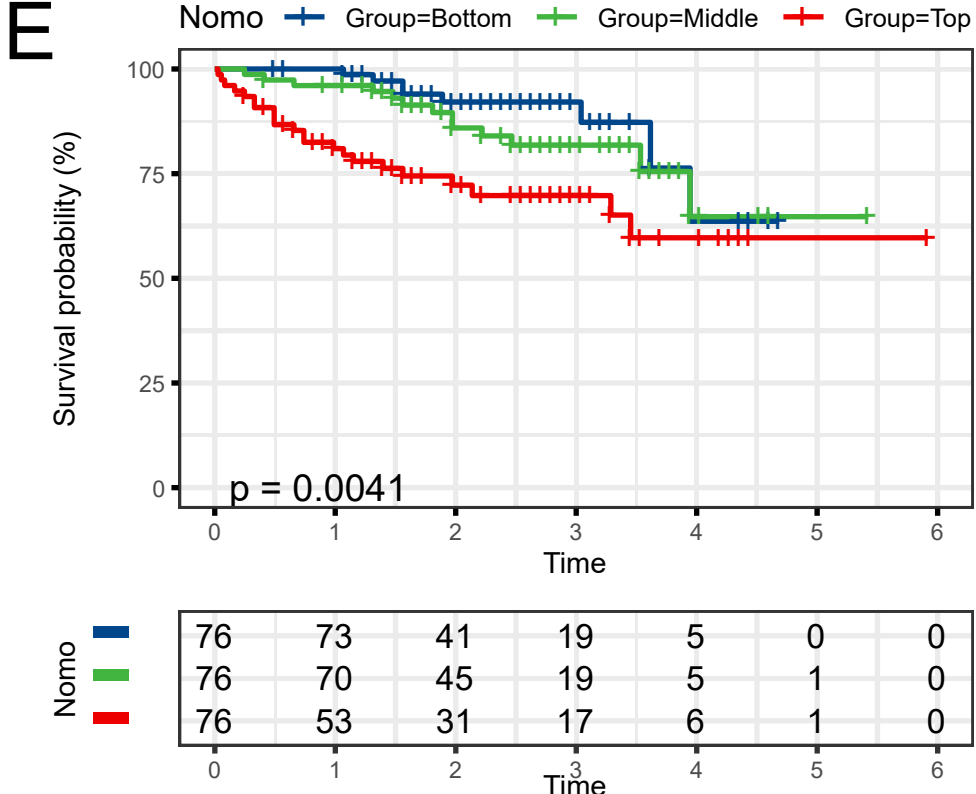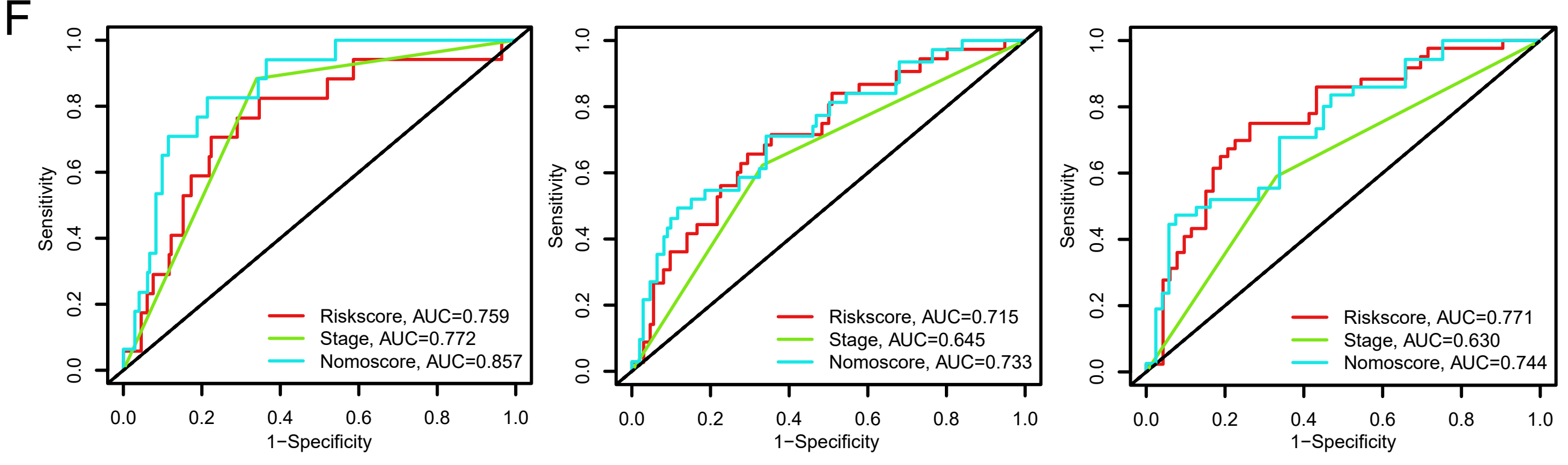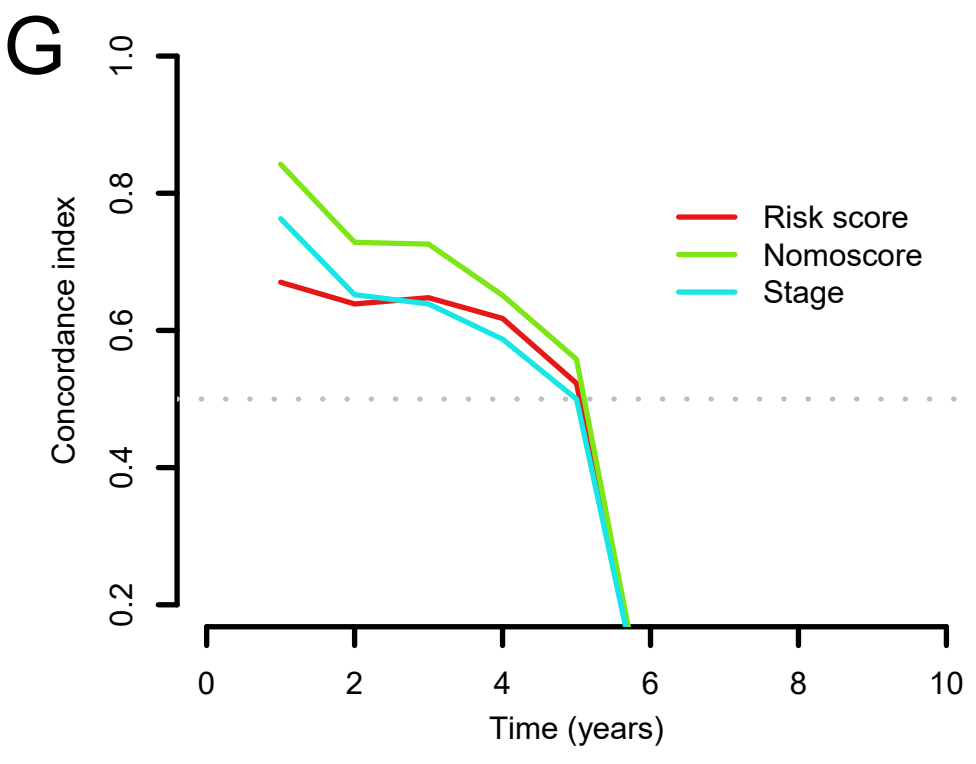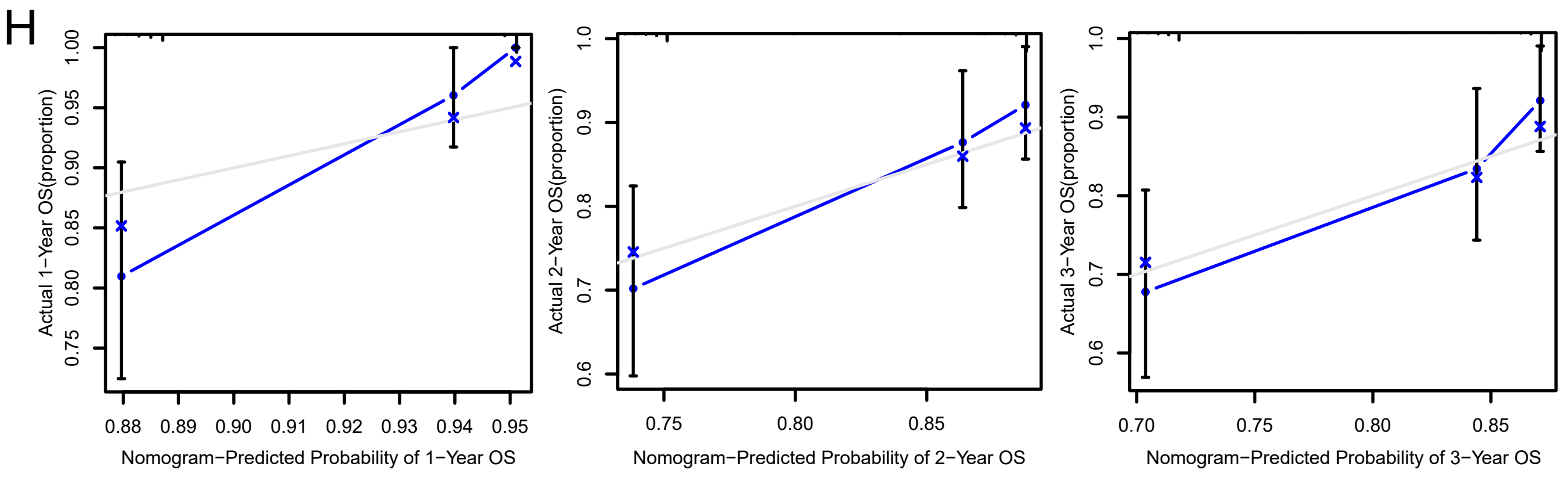

Supplement: Supplementary file 7 [file DataSheet5.PDF]
